# DIAG: A Framework for Evaluating Whole-Genome Amplification Quality in Single-Cell SNV Analysis

**DOI:** 10.3390/biology15100800

**Published:** 2026-05-18

**Authors:** Di Zhang, Mengdong Zhang, Ao Zhang, Siqi Yang, Wenfeng Huang, Tianqi Cao, Xuan Bu, Zhan Liu, Bingjie Chen, Shanjun Deng

**Affiliations:** 1 School of Life Science, Jiaying University, Meizhou 514015, China; zhangd256@mail.sysu.edu.cn; 2 Conservation and Utilization Laboratory of Mountain Characteristic Resources in Guangdong Province, Meizhou 514015, China; 3 MOE Key Laboratory of Gene Function and Regulation, State Key Laboratory of Biocontrol, Innovation Center for Evolutionary Synthetic Biology, School of Life Sciences, Sun Yat-Sen University, Guangzhou 510275, China; zhangmd29@mail2.sysu.edu.cn (M.Z.);; 4 The Guangdong-Hong Kong-Macao Joint Laboratory for Cell Fate Regulation and Diseases, GMU-GIBH Joint School of Life Sciences, Guangzhou Medical University, Guangzhou 511436, China

**Keywords:** single-cell genomics, benchmark of scWGA, quality control, SNV fidelity

## Abstract

Single-cell genomics offers novel insights into genomic heterogeneity within cell populations, reframing our understanding of human development, tumorigenesis, and aging. However, constrained by the picogram-scale DNA templates of individual cells, Whole-Genome Amplification (WGA) remains a necessary precondition. Current quality control frameworks primarily focus on amplification uniformity but fail to capture the molecular independence of DNA amplicons, leading to an overestimation of information content in redundant WGA libraries. Here, we propose the Depth of Independent Amplicons Gauge (DIAG) to accurately quantify the effective number of amplicons derived from the primary template. The robustness of the DIAG was first validated using in silico datasets, revealing that the Depth of Independent Amplicons (DIA) is directly coupled with the precision and specificity of mutation calling. Furthermore, we established an organoid-derived ground-truth to evaluate mutation fidelity in real biological contexts, confirming the practical utility of the DIAG. Our results demonstrate that the DIAG provides a high-fidelity assessment of an individual WGA library without the need for costly external experiments, especially in Single-Nucleotide Variant (SNV) calling. Furthermore, we revealed that traditional uniformity indices, like the Gini index or Kullback–Leibler (KL) divergence, exhibit incongruous fluctuations under down-sampling perturbations. In contrast, the DIA remains a robust and high-fidelity predictor of mutational accuracy, maintaining stability across varying sequencing strategies. Finally, we conducted a systematic comparison of current single-cell Whole-Genome Amplification (scWGA) strategies, providing a standardized benchmarking of diverse technologies for high-resolution single-cell mutation analysis.

## Simple Summary

Every cell has its own unique genetic story, which helps us understand how we grow, how we age, and how diseases like cancer begin. However, a single cell contains such a tiny amount of DNA that it must be “copied” millions of times in a laboratory before it can be studied. This copying process often creates redundant or low-quality data, misleading researchers into believing they have more useful information than they actually do. To solve this, we developed a new tool called the Depth of Independent Amplicons Gauge. This tool acts like a high-precision quality controller, allowing scientists to measure exactly how much unique, reliable genetic information was successfully captured from a single cell. We tested this tool using both computer simulations and real biological samples, proving that it is far more accurate than traditional methods at predicting whether genetic mutations are real or just errors. By developing a standardized method to certify the quality of these genetic amplicons without expensive extra tests, our work helps ensure that medical research and future personalized treatments are based on the most accurate data possible.

## 1. Introduction

Single cells constitute the fundamental operational units of biological systems, exhibiting distinct developmental trajectories and molecular states. Over the past decade, single-cell RNA sequencing has elucidated pseudo-temporal trajectories by capturing static snapshots of transient transcriptomic states. More recently, the emergence of single-cell genomics has provided a longitudinal and evolutionary perspective [1,2,3], enabling the dissection of genetic heterogeneity in developmental biology [4], tumor evolution [5,6,7], organ homeostasis [8], and aging [9,10,11]. However, the extreme rarity and stochastic distribution of the spontaneous mutations necessitate high-fidelity WGA from the femtogram-scale DNA template of a single cell, which is the primary challenge in single-cell genomic research.

To overcome the inherent constraints of single-cell templates, Degenerate Oligonucleotide-Primed PCR (DOP-PCR) and Multiple Displacement Amplification (MDA) utilize degenerate or random primers to achieve non-specific amplification [12,13]. These exponential amplification-based methods, however, inevitably introduce substantial amplification bias, allelic imbalances, and the propagation of errors from the early cycles. To address these limitations, next-generation strategies, like Multiple Annealing and Looping-Based Amplification Cycles (MALBAC) and Linear Amplification via Transposon Insertion (LIANTI), are engineered to preferentially amplify primary templates over daughter strands, leading to a quasi-linear amplification process [14,15]. More recently, Primary Template-directed Amplification (PTA) has refined this approach by utilizing termination bases to effectively suppress the further amplification of daughter strands [16]. These methods aim to ensure that the majority of amplicons derive independently from primary templates, thereby reducing amplicon redundancy and enabling higher-fidelity single-cell genomics analysis. The differences in these amplification strategies are summarized in Table S1, which further incorporates PicoFlex [17], TruePrime [18] and Single-Stranded Sequencing using Microfluidic Reactors (SISSOR) [19].

Beyond technical advancements in amplification strategies, accurately assessing the fidelity of variant calling in single-cell genomic data remains challenging. Conventional quality control frameworks primarily rely on uniformity indicators, like genomic coverage, Gini index, and Lorenz curves, to assess overall amplification performance [20,21,22]. While these indicators are indispensable for copy number variation (CNV) analysis, the fidelity of SNV calling is governed by different characteristics, like allelic imbalance or allele dropout [22]. Relying on uniformity-based indicators for SNV quality assessment creates a methodological mismatch, potentially leading to the overestimation of quality in libraries with high redundancy but low informational complexity. Furthermore, while the Variant Allele Frequency (VAF) density distribution is commonly used to qualitatively reflect allelic imbalance, it remains largely descriptive. Currently, there is a lack of quantitative indicators to decode the underlying amplification mechanisms or to assess the direct impact of such imbalances on SNV calling accuracy for a given dataset. In addition, while benchmarking strategies often employ single-cell clonal expansion to validate the mutation fidelity [4,23,24], additional experimental validation is costly and largely unscalable for most tissues or for routine quality assessment of individual datasets. Consequently, there is an urgent need for a unified, experiment-free quantitative framework to assess the fidelity of variant calling in a single-cell WGA library.

To address this goal, we propose that the DIA provides a fundamentally more rigorous foundation for mutation calling by offering an interpretable and quantitative assessment of allelic imbalance, beyond a descriptive distribution of allele frequency. Theoretically, the reliability of a genomic variant is not merely a function of its allelic fraction among total reads, but of its redundancy-free molecular evidence. Specifically, true variants residing on the primary template will be recurrently captured across multiple independent amplification events. In contrast, stochastic artifacts, like amplified errors, are typically restricted to a single daughter strand and its progenies. Therefore, a higher DIA value reinforces the statistical power of variant calling by ensuring convergent evidence from multiple primary templates, while a lower DIA indicates a larger stochasticity of the library, where early amplification errors propagate more readily and exacerbate allelic imbalances.

In this study, we introduce the DIAG, a unified statistical framework designed to quantify the independent amplification events for a single-cell genomic library. Beyond in silico simulations, we validated the reliability of the DIAG using real-world biological data from single cells and pairwise organoid samples. By formalizing the relationship between amplicon independence and variant reliability, the DIAG establishes a robust, rigorous standard for quantifying the fidelity of mutation calling without the need for extra experiments.

## 2. Materials and Methods

### 2.1. The Mathematical DIAG Framework

Hierarchical sampling model: A two-stage hierarchical sampling model was developed to simulate data from the scWGA stage and the subsequent Next-Generation Sequencing (NGS) library preparation and sequencing stages. For each locus i∈{1, …,N}, let *DIA* denote the depth of independent amplicons.

scWGA stage: For each heterozygote locus with allele *A*/*B*, an amplicon pool is generated. The selection for allele *A* for each amplicon is modeled as a Bernoulli trial with probability *p*. For a standard diploid genome, *p* = 0.5 is assumed for heterozygous loci. However, the model can be generalized to a non-diploid sample (e.g., polyploidy or aneuploid regions in cancer cells) by adjusting *p* to reflect the expected genomic frequency of the allele. The total count of allele *A* amplicons, *K_i_*, follows a binomial distribution as follows:

$$K_{i}∼Binomial(DIA,p)$$

The amplicon allele frequency is defined as *P_i_* = *K_i_*/*DIA*, with

$$E\left[P_{i}\right]=p,\;Var\left(P_{i}\right)=\frac{p(1-p)}{DIA}$$

NGS stage: In the library preparation and sequencing phase, the amplicon pool *P_i_* is further amplified and sampled to produce *M_i_* total reads for locus *i*. We model the number of reads supporting allele *A*, denoted as *y_i_*, as a conditional binomial distribution based on the amplicon frequency *P_i_* as follows:

$$y_{i}|P_{i}∼Binomial(M_{i},P_{i})$$

where *M_i_* is the final sequencing depth at locus *i*. The observed VAF in sequencing data is calculated as $\hat{p_{i}}=y_{i}/M_{i}$.

### 2.2. Derivation of DIA

To estimate DIA, we analyze the total variance of the observed VAF $\hat{p_{i}}$ for a given locus *i* using the Law of Total Variance:

$$Var\left(\hat{p_{i}}\right)=E\left[Var\left(\hat{p_{i}}|P_{i}\right)\right]+Var(E[\hat{p_{i}}|P_{i}])$$

We substitute the properties of the binomial distribution as follows:

Within-pool variance:

$$E\left[Var\left(\hat{p_{i}}|P_{i}\right)\right]=E\left[\frac{P_{i}\left(1-P_{i}\right)}{M_{i}}\right]=\frac{1}{M_{i}}(E\left[P_{i}\right]-E[{P_{i}}^{2}])$$

Given $E\left[{P_{i}}^{2}\right]=Var\left(P_{i}\right)+{\left(E\left[P_{i}\right]\right)}^{2}$, this simplifies to

$$E\left[Var\left(\hat{p_{i}}|P_{i}\right)\right]=\frac{p\left(1-p\right)}{M_{i}}\left(1-\frac{1}{DIA}\right)$$

Between-pool variance:

$$Var\left(E\left[\hat{p_{i}}|P_{i}\right]\right)=Var\left(P_{i}\right)=\frac{p(1-p)}{DIA}$$

Combining these, the theoretical variance is

(1) $$Var\left(\hat{p_{i}}\right)=p\left(1-p\right)\left[\frac{1}{M_{i}}+\frac{1}{DIA}\left(\frac{M_{i}-1}{M_{i}}\right)\right]$$

Using the Method of Moments (MoM), we equate the squared deviation from the mean ${\left(\hat{p_{i}}-p\right)}^{2}$ to the theoretical variance $Var\left(\hat{p_{i}}\right)$:

(2) $${\left(\hat{p_{i}}-p\right)}^{2}\approx E\left[{\left(\hat{p_{i}}-p\right)}^{2}\right]=Var\left(\hat{p_{i}}\right)$$

Rearranging Equations (1) and (2) yields the standardized moment equation:

$$\frac{M_{i}{\left(\hat{p_{i}}-p\right)}^{2}}{p(1-p)}\approx 1+\frac{M_{i}-1}{DIA}$$

Assuming a constant amplification depth across all loci, we aggregate the standardized moment equations across all *N* loci to derive the final DIA estimation.

(3) $$\hat{DIA}=\frac{\sum_{i=1}^{N}\left(M_{i}-1\right)}{\sum_{i=1}^{N}\left[\frac{M_{i}{\left(\hat{p_{i}}-p\right)}^{2}}{p\left(1-p\right)}-1\right]}$$

where $M_{i}$ and $\hat{p_{i}}$ is the sequencing depth and the VAF for locus *i*, respectively, *N* is the total number of heterozygous loci, and *p* is the proportion of the allele.

### 2.3. Implementation of the DIAG Framework

**Figure 1:**
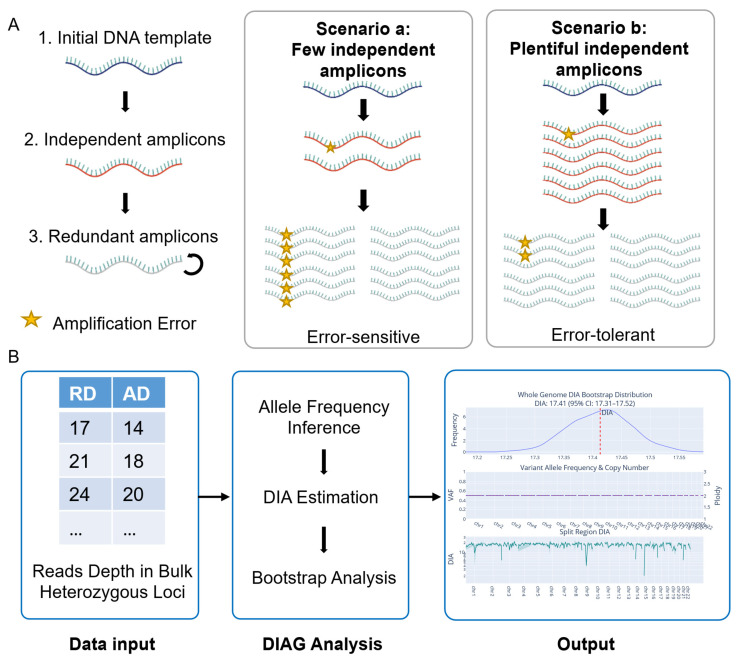
Conceptual foundation and analytical workflow of the DIAG framework. (**A**). Theoretical framework of DIAG. The single-cell amplification process is modeled as a two-stage hierarchical process. Stage 1 involves the generation of independent amplicons directly from the primary DNA template, defining the depth of independent amplicons as DIA, while Stage 2 involves the subsequent amplification of existing amplicons (redundant amplicons). Stars indicate enzyme-induced errors. Scenario *a* illustrates how limited DIA makes the library highly sensitive to early amplification errors, leading to their propagation as false positives. Conversely, Scenario *b* (higher DIA) demonstrates how increased template sampling enables the suppression of such errors through a consensus strategy, where the true genomic signal outweighs stochastic artifacts. (**B**). The workflow of the DIAG statistic framework. The input consists of sequencing count for Reference Depth (RD) and Alternative Depth (AD) at bulk validated heterozygous loci. The central panel delineates the DIAG analytical pipeline, featuring allele frequency inference, DIA estimation by hierarchical model, and robustness validation through bootstrap analysis. The right panel displays a representative HTML output report, providing a comprehensive visualization of the DIA values, VAF distributions, and estimation reliability for a given scWGA sample.

As illustrated in Figure 1B, the DIAG framework is implemented in three major stages. Following data input, we first inferred the VAF required for DIA estimation across specific genomic regions (e.g., individual chromosomes, aneuploidy segments). We then calculated DIA values using Equation (3) for the whole genome. To account for localized amplifications, the genome was partitioned into sub-regions, for which independent DIA estimates were derived. Statistical uncertainty was quantified through bootstrap resampling of loci to generate confidence intervals. Finally, the resulting DIA estimates and inferred VAFs were visualized using interactive plots generated with the Plotly Python library (version 6.5.0; Plotly Technologies Inc., London, UK, https://plotly.com/python/ (accessed on 31 December 2025)).

### 2.4. Evaluation of the DIAG Framework in Simulated Dataset

#### 2.4.1. Simulation in Silico Sequencing Data

**Figure 2:**
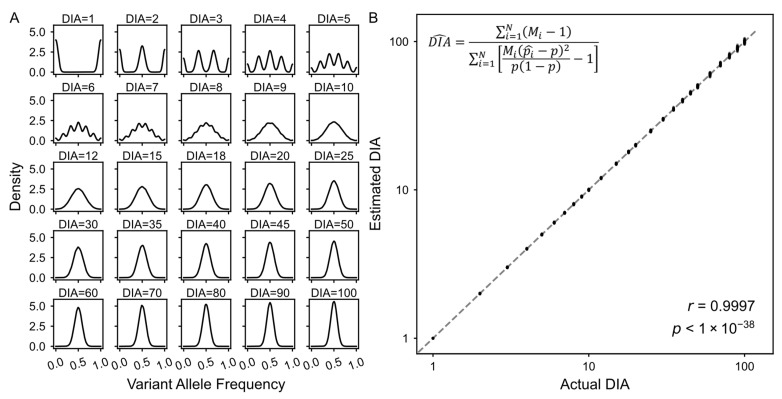
Accurate recapitulation of VAF distributions by DIAG in in silico simulation. (**A**). Density distributions of allele frequencies across increasing DIA values. Subplots are arranged in order of increasing DIA values (ranging from 1 to 100). As DIA increases, the allele frequency distributions converge toward a central peak at 0.5 from a broad distribution, indicating a significant reduction in stochastic allelic variance. All panels utilize Gaussian kernel density estimation (*y*-axis) plotted against allele frequency (*x*-axis). (**B**). Accuracy and linearity of the DIAG framework via the DIAG framework. Evaluation of the DIAG framework using in silico simulated data under the baseline scenario. The *x*-axis represents the ground-truth DIA values, and the *y*-axis represents the estimated DIA values across 100 independent simulation replicates. The dashed diagonal line indicated the y = x identity line, representing a perfect estimation. The accompanying equation defines the method of calculating the DIA value. Pearson’s correlation coefficient (r) and the *p* value obtained from the two-sided correlation test are shown.

To evaluate the accuracy of DIA estimation, we performed in silico simulations mimicking the two-stage scWGA-NGS process. For each heterozygous locus *i* with alleles A and B, we first simulated the scWGA process from the genomic template. The number of amplicons carrying allele A (*K_i_*) was sampled from a binomial distribution:

$$K_{i}∼Binomial(DIA,p)$$

where *p* represents the probability of selecting allele A (set to 0.5 for diploid heterozygous loci). Next, we simulated the NGS process by sampling reads from the resulting amplicon pool. The number of reads supporting allele A (*y_i_*) was sampled from a second binomial distribution:

$$y_{i}∼Binomial\left(M_{i},\frac{K_{i}}{DIA}\right)$$

where *M_i_* represents the total sequencing depth at locus *i*, and the ratio *K_i_*/*DIA* represents the probability based on the amplified VAF. In the baseline scenario, we simulated 100,000 independent loci with a fixed sequencing depth of *M_i_* = 30 and an allele probability of *p* = 0.5. The resulting distribution of simulated allele frequencies is presented in Figure 2A.

#### 2.4.2. Assessing the Accuracy of the DIAG Framework

To validate the estimation framework, we simulated the baseline scenario across a range of DIA values, performing 100 independent replicates for each case. We evaluated the correlation between the estimated values and the ground-truth DIA. Estimation accuracy for each replicate was calculated using the following formula:

$$Accuracy=1-\frac{\left|\hat{DIA}-DIA\right|}{DIA}$$

where $\hat{DIA}$ represents the estimated value and DIA represents the true parameter used in the simulation. The $\hat{DIA}$ is the mean value of the 100 independent replicates.

#### 2.4.3. Assessing the Robustness of the DIAG Framework

To evaluate the robustness of the DIAG framework, we introduced four specific variations separately to the baseline simulation.

To simulate stochasticity in amplification efficiency, we modeled the actual DIA (${DIA}_{actual}$) using two distributions centered around the average DIA (${DIA}_{avg}$):

${DIA}_{actual}\sim N\left(1,\sigma \right)\times {DIA}_{avg}$, with σ ranging from 0 to 1 (*μ* = 1), and coefficient of variation (CV = *σ*/*μ*) ranging from 0 to 1. To ensure biological plausibility, the distribution was truncated to exclude non-positive values, and the ground-truth DIA is adjusted using the following truncated normal distribution formula:

$$E\left[X|X>a\right]=\mu +\sigma \frac{\phi \left(\frac{a-\mu }{\sigma }\right)}{1-\Phi \left(\frac{a-\mu }{\sigma }\right)}$$

where *μ* is the mean of the original distribution, *σ* is the standard deviation, *a* is the truncation point, and ϕ and Φ is the probability density function (PDF) and cumulative distribution function (CDF) of the standard distribution, respectively.

${DIA}_{actual}\sim U\left(1-\delta ,1+\delta \right)\times {DIA}_{avg}$, with relative half-width δ ranging from 0 to 1.

We reduced the sequencing depth (*M_i_*) and the total number of detected loci to simulate insufficient sequencing depth and locus dropout, respectively.

We introduced errors during the scWGA stage. The number of erroneous amplicons (allelic misreadings) was sampled as

$$Errors\sim Binomial\left(K_{i},\varepsilon \right)$$

where *ε* represents the error rate.

We varied the allele probability *p* to simulate chromosomal CNVs and aneuploidy regions.

Each variation was simulated across a range of DIA values, with results averaged over 100 independent replicates for each condition.

#### 2.4.4. In Silico Benchmarking for Single-Cell SNV Calling in DIAG Framework

To evaluate the utility of DIA, we conducted in silico simulations mimicking single-cell SNV calling. We first employed Monte Carlo simulations to estimate the probabilities of specific genotyping errors. By simulating raw sequencing reads, performing genotyping, and comparing the resulting genotypes with a known ground-truth, we quantified the frequency of the following stochastic errors:

Heterozygous Allele Dropout: The stochastic loss of one allele at a true heterozygous site.

Homozygous-to-Heterozygous (Homo-to-Heter) Transitions: The misclassification of a true homozygous site as heterozygous.

Homozygous-to-Homozygous (Homo-to-Homo) Transitions: The misclassification of a true homozygous site as the alternative homozygous genotype.

Using the derived error probabilities (Figure S1), we simulated single-cell SNV calling across the genome. The following metrics were used to assess the accuracy of the DIAG.

True Positive (TP): Correctly identified germline mutations.

False Negative (FN): True mutations that were not detected.

False Positive (FP): Wild-type loci incorrectly identified as mutations.

Allele Dropout (ADO): True heterozygous loci incorrectly called as homozygous.

To further benchmark the SNV calling performance, we calculated sensitivity (recall) and precision as follows:

$$Sensitivity=\frac{TP}{TP+FN}$$

$$Precision=\frac{TP}{TP+FP}$$

For the baseline scenario, the simulation was conducted using a human genome-scale model consisting of 3 × 10^9^ total sites, including 1 × 10^6^ true heterozygous sites and 1 × 10^6^ alternative homozygous sites. SNV calling was assessed across a DIA value scale of 1–100, with polymerase error rates ranging from 1 × 10^−6^ to 1 × 10^−2^.

### 2.5. Evaluation of the DIAG Framework in Biological Dataset

#### 2.5.1. Lung Organoid Culturing and Paired Single-Cell Isolation

In this study, we collected 2 lung bulk tissues from 2 individuals. The lung tissues were separated from donor bodies and digested into a single-cell suspension using Primary Tissue Lysis Buffer (Shanghai JFKR Organoid Biotechnology, Shanghai, China; OrganoPro™, Cat. No. JFKR-NL-100-KIT). Then, we lysed the erythrocyte using Red Blood Cell Lysis Buffer (Solarbio Science & Technology, Beijing, China; Cat. No. R1010) for 3–5 min, removed the debris using a Debris Removal Kit (RWD Life Science, Shenzhen, China; Cat. No. DHDR-5006), and filtered dead cells using a Dead Cell Removal (Annexin V) Kit (STEMCELL Technologies, Vancouver, BC, Canada; EasySep™ Cat. No. 17899). The whole procedure was processed on ice or at 4 °C. Finally, lung organoid formation was initiated by seeding 20–50 thousand live primary cells per 50 μL droplet. The seeding suspension consisted of 70% Matrigel (Corning, Corning, NY, USA; Cat. No. 356231) and 30% specialized culture medium (DMEM/F-12, Thermo Fisher Scientific, Waltham, MA, USA; Gibco™, Cat. No. C11330500BT), supplemented with lung-specific growth factors as previously described [25]. The plate was placed inverted in a culture incubator (37 °C, 5% CO_2_) for 30 min. Then, another 1 mL culture medium was added to the sample, the plate was returned to the culture incubator, and the culture medium was refreshed every 3–4 days.

The samples were resolved from Matrigel when the cell number was sufficient with the Corning Cell Recovery solution (Corning, Cat. No. 354253). After centrifuging at 300× *g* at 4 °C, we discarded the supernatant and resuspended the sample in DMEM-F12 medium. Then, we transferred the single organoid into a 90 mm dish and isolated morphologically complete organoids into separate droplets with the lysis buffer (Thermo Fisher Scientific, TrypLE™ Express Cat. No. 12604013). Then, after washing with DPBS buffer (Thermo Fisher Scientific, Gibco™, Cat. No. 14190144), paired single-cell and multiple-cell clones from the same organoid were placed into separate tubes for later amplification. Our datasets comprise 11 MALBAC-amplified single cells with their 11 corresponding parental organoid samples from P1 donor, and 2 PTA-amplified single cells with their 2 matching organoids from P2 donor. The sample information is shown in Table S2.

#### 2.5.2. WGA and Sequencing

The organoid and corresponding single cell were amplified, followed by MALBAC (Yikon Genomics, Shanghai, China; Cat. No. YK001B) and PTA (BioSkryb Genomics, Durham, NC, USA; ResolveOME™, Cat. No. 100956) commercial kits protocol. The libraries of MALBAC samples are processed using TruePrep Flexible DNA Library Prep Kit and TruePrep Index Kit V2 (Vazyme, Nanjing, China; Cat. No. TD504 and TD202). The bulk genome was extracted by the Tissues Genomic DNA Extraction Kit (Generay, Shanghai, China; Cat. No. S2140715). All the DNA products were purified using the SPRI beads (Beckman Coulter, Brea, CA, USA; Cat. No. B23318). All sequencing libraries were generated and sequenced on the Illumina platform by Haplox and GENEWIZ.

### 2.6. Data Preprocessing and the DIAG Framework

#### 2.6.1. SNV Calling

Whole-genome sequencing data were processed using the nf-core/sarek pipeline (v3.4.2) [26] via Nextflow (v24.04.4) [27] in joint-genotyping mode to enhance variant concordance and detection sensitivity across the cohort. Raw FASTQ reads were quality-trimmed using fastp (v0.23.4) [28] and aligned to the human reference genome GRCh38 (GATK resource bundle) with BWA-MEM2 (v0.7.17) [29]. The mutation calling process followed the GATK standard pipeline [30]. Specifically, post-alignment processing included duplicate marking and base quality score recalibration (BQSR) using GATK4 (v4.5.0.0), with known variant resources from dbSNP (build 146), known indels from GATK Bundle, and Mills and 1000 Genomes gold-standard indels. Variants were called per sample in GVCF mode using Haplotype Caller, consolidated via Genomics DBImport, and jointly genotyped across all samples with Genotype GVCFs. Variant Quality Score Recalibration (VQSR) was applied separately for SNPs and indels using standard GATK training resources (1000 Genomes phase 1 SNPs, Mills gold-standard indels, and dbSNP build 146), with a 90% truth sensitivity threshold applied to retain high-confidence variants.

#### 2.6.2. Validation of the DIAG Framework in PTA and MALBAC

To estimate the number of independent amplification events, we first identified germline heterozygous SNVs by comparing each single-cell (or organoid) sample with its corresponding bulk DNA. The VAFs at these bulk-confirmed heterozygous loci were calculated for each amplified sample and subsequently utilized as the core input for the DIAG framework. The framework processed the VAF distributions to compute the DIA value, a quantitative metric representing the effective template-derived amplicons count for each sample, and then exported the DIA value and confidence intervals in a Hyper Text Markup Language (HTML-based) file.

For comparisons of DIA values across different amplification technologies (MALBAC and PTA), statistical significance was determined using the Wilcoxon rank-sum test.

We selected the SNVs from the gVCF file, filtered by “GQ ≥ 20 and DP ≥ 10”, and calculated the count of SNVs only in autosomes as follows:

TP: genotype 0/1 or 1/1 in the bulk sample, and genotype 0/1 or 1/1 in the amplified sample;

FN: genotype 0/1 or 1/1 in the bulk sample, and genotype 0/0 in the amplified sample;

FP: genotype 0/0 in the bulk sample, and genotype 0/1 or 1/1 in the amplified sample;

True Negative (TN): genotype 0/0 in the bulk sample, and genotype 0/0 in the amplified sample.

Then, we computed sensitivity and precision in the same manner as the simulation.

In this study, somatic mutation (SM) was rigorously identified using a combination of genotype filtering and biological cross-verification. Specifically, a variant was considered a somatic mutation only if it exhibited a homozygous reference genotype (0/0) in the bulk sample and a heterozygous or homozygous alternative genotype (0/1 or 1/1) in both the single-cell sample and its corresponding organoid sample. By requiring the concurrent presence of somatic mutations in both the single cell and the organoid, we implemented a “biological filter” to distinguish authentic biological variations from stochastic amplification artifacts. Since organoids inherit the genomic profile of the founding cell, this overlap significantly enhances the fidelity of our mutation calling. Then, we defined the Signal-to-Noise Ratio (SNR) as the ratio between the number of identified high-confidence somatic mutations and the FP calls.

#### 2.6.3. PTA Data Processing and Uniformity Manipulation

The PTA dataset was down-sampled based on local coverage depth to simulate varying levels of uniformity. Specifically, chromosome 1 was extracted and partitioned into 10 kb windows. For each predefined quantile cutoff c (c in 0.95, 0.75, and 0.5), the read depth in windows exceeding the c-th quantile was capped at that specific threshold. Subsequently, a second round of global down-sampling was performed to normalize the overall mean depth to approximately 20× across all conditions. Coverage profiles for chromosome 1 were visualized in 50 kb bins, with panels organized by sample and down-sampling cutoff. For each processed dataset, five metrics were evaluated: Gini index, KL divergence, DIA inference, precision, and sensitivity.

### 2.7. Systematic Evaluation of the DIAG Framework Across Multiple scWGA Methods

To evaluate the DIAG framework across multiple platforms, we conducted a comprehensive benchmarking consisting of 36 single-cell datasets spanning six major scWGA technologies.

Publicly Sourced Datasets: We downloaded 18 single-cell libraries’ sequencing data from Chen et al. [15], which included samples for DOP-PCR (*n* = 3), LIANTI (*n* = 3), MALBAC (*n* = 3), MDA (*n* = 6), and PicoPlex (*n* = 3). In addition, we also incorporated PTA datasets (*n* = 5) from Pena et al. [16].

In-house Datasets: In-house single-cell genomic data comprised 11 cells amplified using MALBAC and 2 cells amplified using PTA.

The sample data information is shown in Table S3.

All raw datasets were processed through a unified pipeline to ensure comparability. The DIA values, germline SNV definition, mutation count, sensitivity, and precision are calculated as mentioned before; the correlation analysis used Spearman’s method.

## 3. Results

### 3.1. Theoretical Framework of the DIAG

scWGA is essentially a process of allelic information transfer from minimal primary templates to an NGS library. Within this workflow, the obtained reads are derived from two distinct populations of amplicons (Figure 1A). The initial products directly and independently derived from the primary templates are termed independent amplicons, whereas the remaining amplicons, produced through the further amplification of existing products, are redundant. The DIA represents the effective number of these independent amplicons, indicating the molecular complexity of the library.

For single-cell genomic analysis, both the enzymic error rate and the DIA would dominantly dictate the reliability of variant detection. The former determines the frequency of stochastic errors, a well-recognized intrinsic property of specific WGA strategies. The latter, however, has been frequently overlooked in previous studies. A lower DIA leads to an overrepresentation of redundant amplicons, allowing polymerase-introduced errors to propagate and become indistinguishable from true variants (Figure 1B). In contrast, a higher DIA dilutes the impact of such stochastic errors by ensuring they occur in only a small fraction of independent amplicons, while true mutations remain consistently represented. This allows artificial errors, which are unlikely to recur at the same locus across independent templates, to be effectively filtered using a consensus strategy (Figure 1B). Therefore, given a comparable enzymatic error rate, DIA provides a direct and standardized assessment of amplification performance for any scWGA library.

To derive DIA, we modeled the scWGA and NGS workflows as a two-stage hierarchical stochastic process. Utilizing variance decomposition and the MoM, we derived a formal relationship to estimate DIA based on the VAF distribution across heterozygous loci (Methods). To ensure the robustness of our framework across the heterogeneous genomic landscape, the DIAG supports partitioned analysis based on genomic features, such as GC content and CNV, allowing for the quantification of DIA within specific genomic bins (10M). Furthermore, we implemented bootstrap resampling of loci to quantify statistical uncertainty and generate confidence intervals. This results in a comprehensive, user-friendly quality assessment report for each single-cell library (Figure 1B).

### 3.2. Accurate Recapitulation of VAF Distributions by the DIAG in In Silico Simulation

To validate the performance of the DIAG framework, we performed in silico simulations mimicking the two-stage scWGA-NGS process. For each locus, we first sampled the alleles of independent amplificons from the genomic template, followed by sampling sequenced reads from this independent amplificon pool (Methods). Then, we tested the sensitivity of the VAF distribution to variations in DIA (Figure 2A). As expected, lower DIA values introduced significant sampling variation at the amplification stage, manifesting as highly dispersed VAF distributions and prominent pseudo-homozygous peaks. Conversely, as DIA increased, the distributions became increasingly concentrated around the expected heterozygote frequency of 0.5. These results indicate that different DIA values can represent a distribution that intuitively reflects allele frequencies in heterozygous loci.

The simulated sequencing data were evaluated using the mathematical DIAG framework (Figure 2B). Under a baseline scenario under 30× sequencing depth across 100,000 loci, the estimated DIA values exhibited a nearly perfect correlation with the predefined ground-truths (Figure 2B; r = 0.9997, *n* = 100, *p* < 1 × 10^−38^). Across multiple replicates, the estimation accuracy consistently remained between 94% and 100% (Figure S2). Therefore, the DIAG accurately recapitulates the VAF distributions, providing a potential solid analytical framework.

### 3.3. Robustness of the DIAG Framework Across Technical and Biological Variables

We then systematically evaluated the robustness of the DIAG framework under various technical and biological perturbations, including non-uniform DIA distributions, varying sequencing depths, genomic data scales, intrinsic enzymatic error rates, and pervasive aneuploidy.

**Figure 3:**
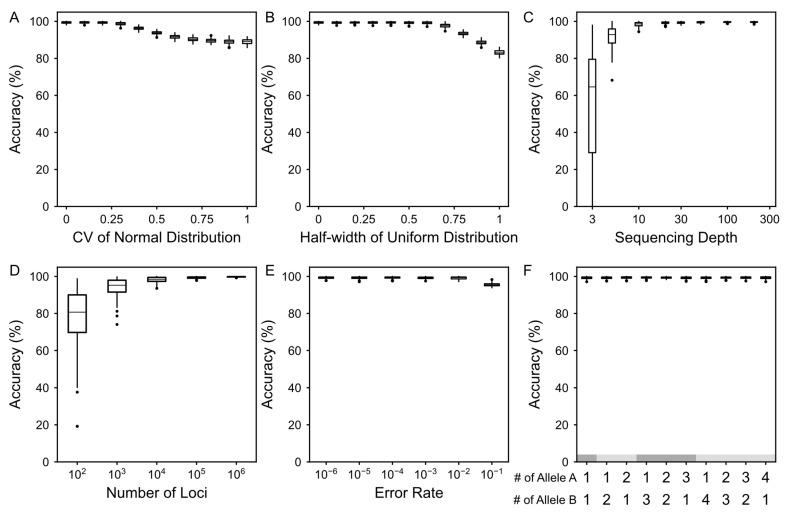
Robustness of the DIAG framework across varying experimental and biological parameters. All evaluations were performed using 100 independent replicates with a ground-truth DIA of 30. (**A**). Performance of DIAG under varying standard deviation of the normal distribution. Boxplots represent estimation accuracy across a range of CV from 0 to 1, indicating the stability of the DIAG even when the distribution of independent amplicons deviates from the mean. (**B**). Performance of the DIAG under varying interval widths of the uniform distribution. Accuracy is shown for different relative half-width (δ) ranging from 0 to 1. High accuracy is maintained under broad uniform interval sampling, demonstrating resilience to local amplification bias. (**C**). Sensitivity to sequencing depth. Accuracy is compared across a broad range of sequencing depths from 3× to 200×, establishing the minimum recommended depth for reliable estimation. (**D**). Impact of effective loci count. Accuracy of estimation is maintained as the total number of loci scales from 1 × 10^2^ to 1 × 10^6^. The estimation remains highly accurate with a limited number of loci. (**E**). Robustness to amplification errors. Accuracy of estimation is shown for error rate ranging from 1 × 10^−6^ to 0.9, demonstrating that the DIA metric is independent of enzymatic fidelity. (**F**). Robustness to CNVs. Accuracy of estimation remains stable across varying ploidy levels from 2 (diploid) to 5 (pentaploid). The *x*-axis illustrates allelic configurations across simulated genomic loci, with the underlying gray shading distinguishing the varying total ploidy levels, ranging from 2 (diploid) up to 5 (pentaploid). Across all panels, the center line indicates the median, box bounds represent the upper and lower quartiles, and the whiskers extend to 1.5× Inter-Quartile Range (IQR). Outliers are shown as individual points.

First, the DIAG demonstrates high tolerance to amplification preferences across the genome. By modeling DIA as a distribution rather than a fixed constant, we observed that DIA estimates remained stable under various distributional assumptions. Under normal distributions, the accuracy of estimation is relatively stable across different standard deviations (Figure 3A and Figure S3A). Similar results were obtained using a uniform distribution (Figure 3B and Figure S3B), further indicating that the DIAG can robustly recover DIA signals even in genomic regions prone to severe amplification bias, such as GC-enriched or repetitive sequences.

Second, we assessed the impact of sequencing depth and the number of informative loci on the estimation accuracy. The DIAG shows rapid saturation with respect to sequencing depth and maintains high fidelity even under low-coverage conditions (Figure 3C and Figure S3C), achieving an average accuracy of 91.8% at 5× coverage. Furthermore, the DIAG exhibits remarkable insensitivity to the number of informative loci (Figure 3D and Figure S3D), suggesting its potential for application in high-throughput but low-pass sequencing libraries or region-specific genomic assessments. Collectively, these results suggest that a minimum empirical requirement for reliable single-cell quality control is 5× coverage with at least 1000 informative sites.

In addition, we confirmed that DIA inference is decoupled from the enzymatic error rate. Conceptually, these two factors are independent because the enzymatic error rate determines the frequency of primary amplification errors, whereas the DIA dictates how these errors are represented in the final sequencing library. Our results confirm this by showing that the DIAG is highly insensitive to the underlying enzymatic error rate (Figure 3E and Figure S3E). Specifically, DIA inference maintains a stable performance even at an error rate as high as 0.1, achieving an average accuracy of 95.5%. This underscores that DIA captures the physical independence of amplicons rather than the biochemical fidelity of the polymerase.

Furthermore, the DIAG remains robust within complex biological contexts characterized by pervasive aneuploidy. To account for the CNV typical of tumor cells, we adjusted the preset allele frequency *p* to reflect local ploidy. Notably, the DIAG accurately recovered the number of independent amplicons across diverse copy number states (Figure 3F and Figure S3F), demonstrating its reliability for assessing atypical single-cell datasets with an unstable genome. Collectively, these results establish the DIAG as a robust and reliable tool, unconfounded by common technical artifacts or complex biological contexts.

### 3.4. DIA as a Compraehensive Indicator of SNV Calling Fidelity

The ultimate utility of a quality control metric lies in its capacity to predict downstream analytical performance. Therefore, we systematically evaluated the relationship between DIA and downstream mutation calling performance. Using an in silico dataset with a known ground-truth, we benchmarked DIA against widely used critical performance indicators, including TP count, ADO, FN count, FP count, precision, and sensitivity, under varying enzymatic error conditions. This benchmarking effort aims to bridge the gap between abstract library complexity and the concrete reliability of somatic mutation discovery, serving as a quantitative basis for setting quality thresholds in single-cell genomics analysis.

**Figure 4:**
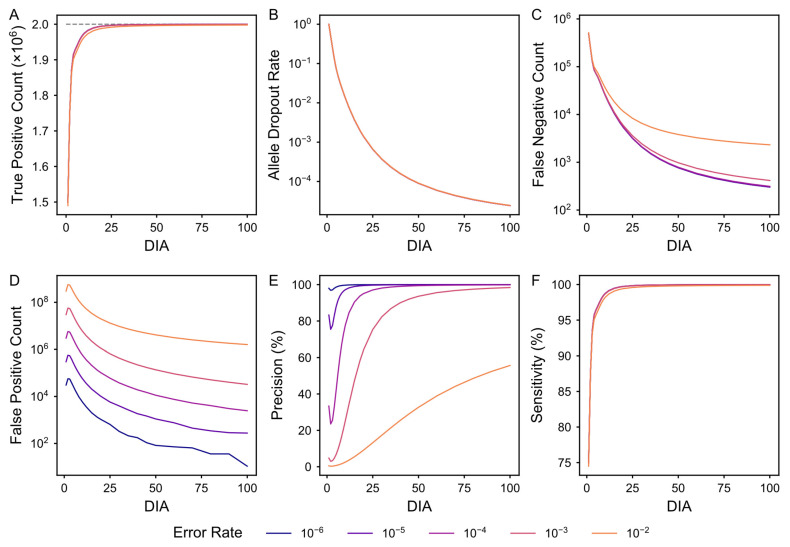
Benchmarking of SNV calling performance across DIA values and enzymatic error rates. All metrics were evaluated using in silico simulated data under a baseline scenario mimicking the human genome (3 × 10^9^ total loci with 1 million heterozygous and 1 million homozygous germline mutation loci). (**A**). TP count. The recovery of TP improves rapidly and reaches saturation as DIA increases; the gray dashed horizontal line indicates the ground-truth positive count. The dashed horizontal line indicates the theoretical upper bound for TP. (**B**). ADO rate. The rate shows a consistent exponential decay across all simulated error conditions as DIA increases. (**C**). FN count. The metric decays exponentially as DIA increases, and higher baseline error rates result in significantly higher plateaus. (**D**). FP count. This metric shows an inverse relationship with DIA and is highly sensitive to the error rates, spanning several orders of magnitude. (**E**). Precision. Precision shows a positive relationship with DIA. (**F**). Sensitivity. Performance increases rapidly and stabilizes near 100% for all error rates as DIA increases. Across all panels, SNV calling was assessed across a DIA range of 1–100; solid lines represent the mean performance, with colors indicating polymerase error rates ranging from 1 × 10^−6^ to 1 × 10^−2^.

First, DIA is the primary determinant of recovered TP variants and ADO mitigation. As DIA increases, the recovery of true variants improves rapidly and reaches saturation (Figure 4A). Conversely, ADO is drastically mitigated at higher DIA levels, as the probability of failing to capture a specific allele decreases exponentially with the accumulation of independent amplicons (Figure 4B). Notably, as DIA dictates the probability that a variant is physically captured and detected, we found that the enzymatic error rate is decoupled from these two indicators.

Second, high DIA suppresses false variants through a molecular consensus mechanism. We found that both FN and FP counts are negatively correlated with DIA (Figure 4C,D). At low DIA levels, stochastic errors introduced during early amplification cycles can become overrepresented, making them indistinguishable from true biological variants and inflating the FP count. However, as DIA increases, these random errors are effectively suppressed through the consensus of multiple independent amplicons derived from the same template. While higher enzymatic error rates increase the baseline of errors, a high DIA enables a robust filter to suppress these stochastic artifacts, significantly enhancing precision and sensitivity (Figure 4E,F).

In addition, by conducting our analysis of heterozygous and homozygous loci, two distinct benchmarking profiles were found (Figure S4). Specifically, ADO was identified as the primary source of error at heterozygous sites, representing a fundamental sampling failure. In contrast, polymerase-induced amplification errors dominated at homozygous sites, where the absence of a second biological allele renders the system more vulnerable to propagated artifacts. Consequently, while metrics such as TP and ADO are governed almost exclusively by DIA, the FP rate is co-modulated by both DIA and intrinsic enzymatic fidelity (Figure S5). Collectively, our results demonstrate that DIA serves as a robust and comprehensive predictor of scWGA fidelity, offering a unified metric to evaluate the quality of single-cell libraries.

### 3.5. Validation of the DIAG Framework in Real Biological Scenarios

**Figure 5:**
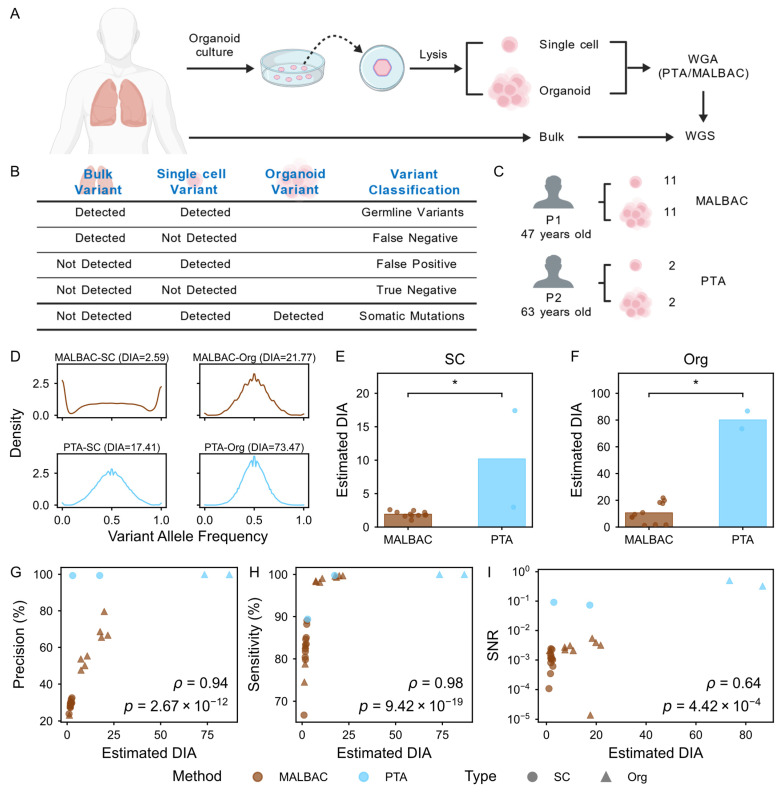
Validation of DIAG in data from PTA- and MALBAC-amplified single cells and organoids. (**A**). Experimental workflow for paired scWGA analysis. Schematic illustration of the pipeline for scWGA derived from primary organoids and their corresponding paired single-cell samples. The samples are processed using either PTA or MALBAC. Bulk sequencing of the same donor material served as the ground-truth control for germline heterozygous loci confirmation. (**B**). Classification of SNVs. A schematic representation of the definitions used within the analysis framework. Metrics include TP, FN, FP, and TN for germline variants and SM. (**C**). Summary of sample information. The table summarizes donor age, paired sample size, and the scWGA strategies employed for each group. (**D**). Representative VAF density distributions. Density plots of VAF at bulk-validated heterozygous sites for the highest DIA samples. The sharper peak at 0.5 reflects the superior template sampling of high-DIA libraries. (**E**,**F**). Comparison of DIA values between MALBAC and PTA. A quantitative comparison reveals that DIA values are consistently higher in PTA-amplified samples compared to MALBAC-amplified samples, both in single cells and in organoids. Bars represent the mean DIA values for each group. Statistical significance was determined using the Wilcoxon rank-sum test (* *p* < 0.05). (**G**,**H**). Correlation between germline variant calling performance and DIA. Spearman’s correlation demonstrates the robust relationship between DIA values against germline SNV precision (**panel G**) and sensitivity (**panel H**). High DIA values consistently correspond to superior calling fidelity. (**I**). Correlation between SNR of somatic mutations and DIA. Spearman’s correlation demonstrates the relationship between the SNR of detected somatic mutations and DIA values. The results indicate that higher DIA improves the reliability of rare somatic variant discovery. Panels (**A**–**C**) were drafted on the Generic Diagramming Platform (GDP) [31]. Panels (**C**–**G**) share the same figure legend; the color represents the method, and the shape represents the sample type. Spearman’s correlation coefficient (ρ) and the *p* value obtained from the two-sided correlation test are shown.

To validate the practicality of the DIAG framework in real biological scenarios, we employed a human organoid culture system as a controlled ground-truth model (Figure 5A). Our experimental design was guided by three key considerations. First, we designed two different biological scales, including single-cell and pairwise organoid samples. Compared to single-cell samples, multicell organoids provide a significantly larger pool of initial genomic templates and thus possess theoretically higher DIA values. Second, by performing pairwise comparisons between single cells and their corresponding organoids, we established a biological gold standard to accurately distinguish true mutations from technical noise, enabling a rigorous assessment of sensitivity and precision (Figure 5B). Third, we evaluated two distinct WGA methodologies, including MALBAC and PTA (Figure 5C). The former is a classic method designed for uniform amplification and the latter is a leading-edge technology that effectively suppresses the re-amplification of daughter strands via termination bases. This multiscale approach allowed us to comprehensively evaluate the DIAG across diverse biological scenarios.

First, we examined the VAF density distributions at heterozygous germline sites. We found that the Allele Frequency Spectrum (AFS) in organoid samples was more tightly centered around the theoretical heterozygous frequency of 0.5 compared to the distributions observed in single cells (Figure 5D and Figure S6). We examined the germline SNV count using the definitions shown in Figure 5B (Figure S7). Quantitatively, the DIAG successfully captured the intrinsic differences in initial template abundance, with single cells exhibiting an average DIA of 10 (ranging up to 17.41), while organoid samples achieved an average DIA of 80 (ranging up to 86.75) (Figure 5E,F). Considering the limited sample size of our PTA cohort (*n* = 2), which may constrain the overall statistical power, we incorporated additional independent datasets, including five PTA-amplified and three MALBAC-amplified single cells. Crucially, the results show that there is a trend where PTA exhibited higher DIA than MALBAC within our evaluated datasets (Figure S8). This demonstrates that the DIA metric successfully quantifies the allelic imbalance that is otherwise only qualitatively visible in AFS plots.

We further assessed the capability of the DIAG to predict library fidelity by performing correlation analyses between DIA and standard performance indicators. Our results revealed that DIA values are positively correlated with both precision (Figure 5G, ρ = 0.94, *p* = 2.67 × 10^−12^) and sensitivity (Figure 5H, ρ = 0.98, *p* = 9.42 × 10^−19^), demonstrating their power as quality evaluation in scWGA library quality.

Beyond germline variants, the pairwise organoid controls provided an exploratory opportunity to evaluate the relevance of DIA to somatic mutation discovery. The somatic mutations in this study are defined as the variants that were not detected in the bulk sample but were detected both in the single-cell sample and the organoid sample (Figure 5B). The count of somatic mutations detected in 13 single cells is shown in Figure S7. In fact, the absolute number of detected somatic mutations is primarily determined by the inherent biological characteristics of the sample, so it is essential to establish a metric to gauge the discovery potential across different libraries. Consistent with this requirement, we observed a positive trend between the SNR and DIA (ρ = 0.64, *p* = 4.42 × 10^−4^; Figure 5I). We should note that the organoid samples in this study were also influenced by the amplification process, leading to a more moderate correlation compared to that observed for germline variants.

Nevertheless, our findings imply that DIA serves as a useful reference for assessing the potential for somatic mutation detection in the WGA library. Taken together, our results demonstrate the robust capability of the DIAG framework in real biological datasets. Specifically, DIAG-based inferences are consistent with experimental variables, accurately reflecting both template abundance and the intrinsic performance of distinct WGA methods. Crucially, the DIAG enables these quality assessments without the need for costly and unscalable validation experiments, ensuring its broad applicability to a specific single-cell genomic dataset.

### 3.6. Cross-Method Benchmarking of scWGA Fidelity via DIAG

While traditional uniformity-based indicators are primarily valuable for CNV analysis, we utilized the DIAG framework to conduct a comprehensive benchmarking of several widely used scWGA methods in public datasets [15,16]. Our analysis revealed several key findings regarding technological performance.

**Figure 6:**
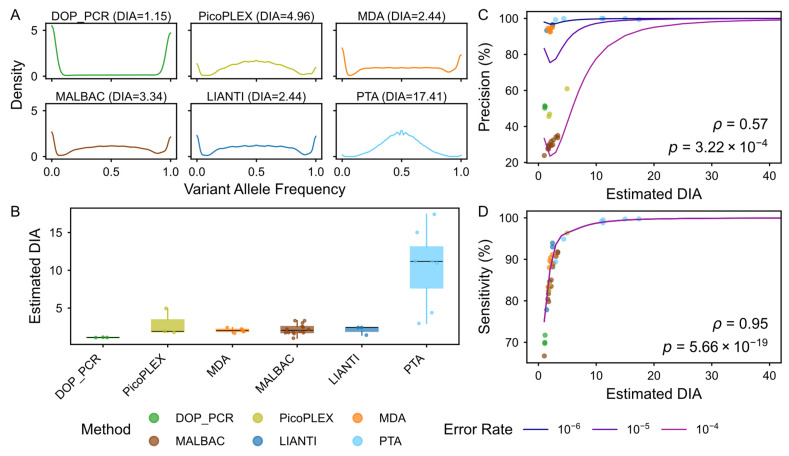
Systematic comparison of DIA in multiple scWGA methods. (**A**). Representative VAF density distributions across diverse scWGA methods. Density plots of VAF at bulk-validated heterozygous loci for the highest DIA samples within six distinct methods (Chen et al., Pena et al., and this study). The sharper peak at 0.5 reflects the superior template sampling of high-DIA libraries. (**B**). Distribution of DIA values for multiple scWGA methods. Comparison of DIA values across various technologies. The points represent DIA values for each single-cell sample. Boxplots indicate the distribution quartiles, with the central line representing the mean value for each method, highlighting the superior template utilization of PTA. (**C**,**D**). Correlation between germline variant calling performance and DIA in single-cell datasets. Spearman’s correlation analysis of DIA values against precision (**C**) and sensitivity (**D**) of germline variants across all tested datasets, indicating that DIA is a universal, platform-independent predictor of variant calling fidelity. The line color in panel **C** and panel **D** represents the benchmark of the simulation at different error rate levels. Panels (**A**–**D**) share the same color legend, representing different scWGA methods.

First, PTA consistently outperformed all other methods in both VAF density distributions and DIA estimations (Figure 6A,B and Figure S9). Early-stage methods, such as DOP-PCR, tended to exhibit low DIA values (averaging 1.12, up to 1.15), reflecting minimal molecular independence and high amplicon redundancy. In contrast, PTA achieved an average DIA of 10.44 (up to 17.41), demonstrating its superior capacity to maintain a diversity of primary genomic templates during amplification.

To further characterize how these DIA variations impact downstream SNV calling fidelity, we integrated DIA estimations with the intrinsic enzymatic fidelity of each method (Figure 6C,D). This further assessment revealed that PTA achieved the highest precision (99.8% on average, up to 99.97%), followed by LIANTI at 95.4% (up to 96.7%). Furthermore, the SNV calling sensitivity of PTA reached an average of 97.3% (up to 99.8%). As summarized in Table 1, PTA consistently outperforms alternative methods in both precision and sensitivity, ensuring reliable single-cell variant discovery.

From another perspective, the DIAG was specially designed to account for genomic heterogeneity, and the framework provides subregion-level DIA estimations in the output report, enabling researchers to dissect variance across distinct genomic features, such as GC content and aneuploidy. To validate this, we examined the relationship between DIA and GC content across 36 single-cell datasets. Our results show that DIA remains largely independent of GC content for the majority of cells (27/36, Figure S10), showing that the DIA effectively captures the complexity of the WGA library despite local genomic heterogeneity. Notably, we observed that certain methods exhibit higher sensitivity to genomic features, such as LIANTI and PTA, and subtle correlations with GC content in specific cells. For instance, a decrease in DIA was observed in high-GC regions for two LIANTI-amplified cells (ρ = −0.64, *p* = 2.11 × 10^−27^ and ρ = −0.61, *p* = 2.09 × 10^−24^, Figure S10). This result suggests that the degree of GC sensitivity varies significantly between amplification chemistries, and the DIAG is capable of pinpointing such localized technical biases. Furthermore, the integration of CNV-aware partitions within DIAG ensures that these principles remain applicable to samples with complex karyotypes. Ultimately, the DIAG enables the localized quantification of WGA quality by automatically partitioning the genome into 10 Mb bins. This granular approach identifies technical variance across heterogeneous genomic regions, ensuring reliable quality assessment even in samples with complex biological contexts.

### 3.7. Decoupling of Traditional Metrics from SNV Calling Fidelity

**Figure 7:**
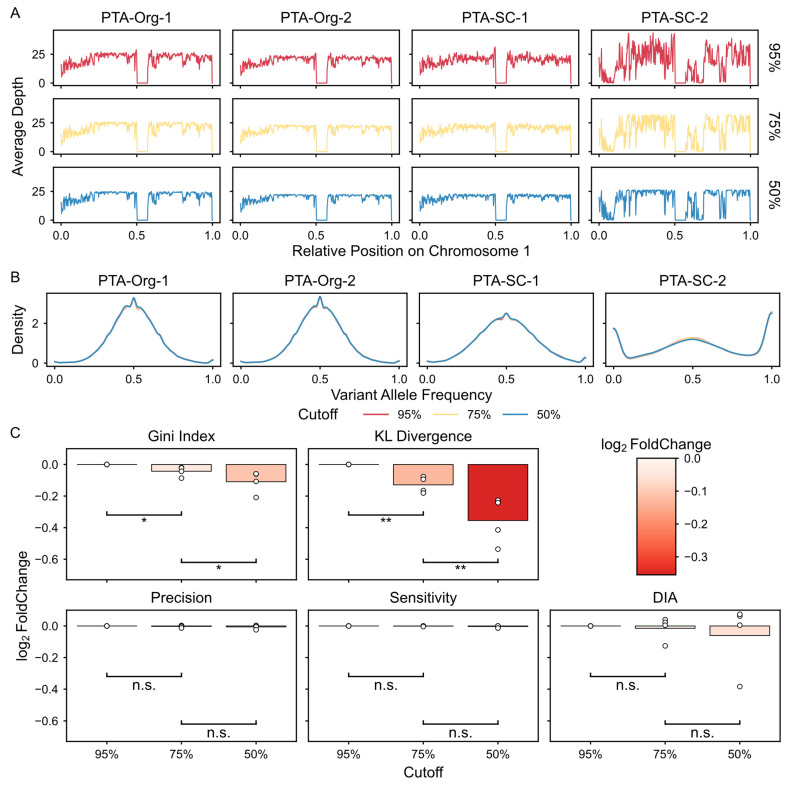
Decoupling of classic uniformity metrics from SNV calling fidelity. (**A**). Coverage across chromosome 1 in 50 kb bins. Panels are organized by sample (columns) and down-sampling cutoff (rows). The *y*-axis shows the mean depth of coverage (X), and the *x*-axis denotes the relative position on chromosome 1. Data are shown for three down-sampling levels as attached on the right. Color represents the cutoff used for clipping during down-sampling. (**B**). The density distribution of VAFs under different cutoffs. Lines are colored according to panel A. (**C**). Panels show specific indicators as labeled above. The *x*-axis represents the down-sampling cutoff, and the *y*-axis indicates the log_2_ fold changes in each sample compared to the baseline at cutoff = 0.95. Significance levels from a one-tailed pairwise *t*-test are indicated: n.s., non-significant; * *p* < 0.05; ** *p* < 0.01.

We further evaluated the relationship between DIA and traditional metrics for the scWGA library. Analysis reveals that DIA remained independent of sequencing depth across five of the six WGA methods (Figure S11). The only exception was DOP-PCR, which lacked sufficient statistical power due to its limited sample size (*n* = 3). Furthermore, while a negative trend was found between DIA and the conventional uniformity metric, like the Gini index (Figure S12), we inferred that the relationship is phenomenological rather than functional. Because the DIA captures the depth of independent amplicons, which inherently determines the ultimate genomic uniformity, it is conceptually distinct from coverage-based metrics. Unlike traditional uniformity measures that only describe the final sequencing distribution, DIA serves as an intrinsic indicator of the initial amplification complexity, effectively capturing how WGA chemistry interacts with genomic heterogeneity, offering a more fundamental assessment of library fidelity. To validate this, we conducted a down-sampling analysis in the same library to evaluate the performance between DIA and the traditional uniformity metric. Crucially, as shown in down-sampling analysis in PTA datasets (Figure 7A,B), uniformity-based metrics such as the Gini index and KL divergence exhibited significant shifts as sequencing depth was reduced by artificial clapping (Figure 7C, *p* < 0.05 and *p* < 0.01, respectively). Furthermore, both precision and sensitivity showed no significant changes under these conditions, confirming that DIA is decoupled from sampling-induced uniformity fluctuations.

Taken together, these results suggest that DIA is a more robust and effective indicator for WGA quality assessment than traditional metrics.

## 4. Discussion

In this study, we introduced the DIAG, a unified statistical framework that redefines quality control for single-cell genomics by redirecting the analytical focus from global uniformity to locus-specific molecular independence. Unlike conventional uniformity-based indices, the DIAG estimates the number of independent amplicons derived directly from primary templates, which is the fundamental determinant of single-cell mutation fidelity. Specifically, the DIAG conceptualizes WGA and NGS as a two-stage hierarchical stochastic sampling process, each characterized by nested binomial distributions. This enables the construction of a comprehensive probabilistic model to rigorously estimate the DIA. Based on the analyses of in silico datasets under diverse perturbations, our results demonstrate that the DIA serves as a robust indicator of the precision and specificity of variant calling, even in complex biological scenarios.

We further demonstrated the practicability of the DIAG using real biological datasets. Using organoid-derived clones as a positive control, we showed that the precision and specificity of mutations are highly correlated with the estimated DIA. Notably, a particular strength of the DIAG is its reference-free nature, which enables intrinsic quality assessment without the need for costly, unscalable experimental controls like single-cell clonal expansion. While previous benchmarking relied on these ground-truth positives to validate specific WGA strategies, such methods are impractical for high-throughput research. The DIAG bypasses this bottleneck by directly quantifying the complexity of each library.

Furthermore, the DIAG effectively captures amplification efficiency across diverse genomic regions. It is well established that GC content dictates the melting temperature and secondary structure stability of DNA templates. High-GC regions increase the energy required for strand separation, potentially leading to stochastic polymerase stalling and reduced processivity during the denaturation and annealing phases of PCR. Conversely, extremely low-GC regions are prone to non-specific priming. While global estimation remains a robust and necessary standard for general analysis, localized genomic features including GC content and CNVs can drive imbalances in amplification across the genome. In scenarios where samples exhibit high regional heterogeneity, the partition-based framework of DIAG provides a valuable alternative by directly reflecting regional amplification efficiency. Rather than viewing these genomic features as confounding variables to be modeled out, this approach treats them as mechanistic drivers, utilizing the DIAG to provide a more precise lens into regional processivity. In addition, the DIAG accounts for amplification imbalance across diverse genomic regions, maintaining a consistent performance even in regions of genomic gain or loss. This characteristic enables the transition from genome-wide descriptive assessment to locus-specific probabilistic modeling, allowing for the high-resolution filtration of stochastic artifacts and ensuring reliable single-cell genomic analysis. With the growing availability of single-cell WGA datasets, we anticipate that the DIAG will become an essential foundation for resolving the plasticity of human development, the clonal origins of tumorigenesis, and the intricate population dynamics of cellular aging.

Data integration from multiple laboratories introduces confounding variables, such as heterogeneous cell types, diverse donor backgrounds, and different sequencing platforms. To enhance the performance of the DIAG in future releases, several technical avenues remain to be explored. First, while the robustness of DIA estimation has been well demonstrated in standard cell-by-cell WGA datasets, it requires approximately 1000 informative heterozygous sites to ensure the output reliability, limiting its utility in ultra-low-coverage samples or regions with extensive loss of heterozygosity. Future optimizations could incorporate disequilibrium information to aggregate sparse local molecular signals for low-quality samples or high-throughput, low-coverage libraries (e.g., DEFND-seq [37]). Second, while the DIAG is able to effectively capture regional amplification variation, the requirement of 1000 informative heterozygous sites for high-confidence estimation limits further genomic subdivision. Consequently, in regions with low SNV density, the method is unable to split the genome into smaller segments, limiting the achievable resolution. Specifically, we have provided an optional analysis in the DIAG framework to control for ploidy-driven biases. In future versions, we anticipate refining this into a joint probabilistic model that simultaneously accounts for multiple genomic covariates, enabling a more robust and fully automated analysis. Third, by leveraging locus-specific DIA values, a likelihood-based scoring system could be implemented to statistically distinguish high-confidence mutations from stochastic background noise.

## 5. Conclusions

In conclusion, by shifting the analytical paradigm from global uniformity to molecular independence, the DIAG establishes a rigorous standard for single-cell genomics without extra experiments. This framework not only enables the systematic benchmarking of diverse WGA strategies but also provides robust, in situ quality control for individual single-cell datasets. We provide the DIAG as a user-friendly package for board research applications.

## Figures and Tables

**Table 1:** Comparative performance of six scWGA methods.

scWGA Method	Enzyme	Amplification Error Rate (Order of Magnitude)	DIA	Precision (%)	Sensitivity (%)
DOP-PCR	DNA polymerase	1 × 10^−4^~1 × 10^−5^ [32]	1.12 ± 0.02	50.9 ± 0.6	70.5 ± 1.1
PicoPLEX	Strand-displacement enzyme, DNA polymerase	1 × 10^−4^~1 × 10^−5^ [32,33]	2.90 ± 1.46	51.1 ± 8.5	87.6 ± 7.6
MDA	phi29	1 × 10^−6^ [34]	2.05 ± 0.25	93.9 ± 1.1	88.6 ± 2.8
MALBAC	Bst; Taq DNA polymerase	1 × 10^−4^ [35,36]	2.20 ± 0.66	30.1 ± 3.0	84.2 ± 6.5
LIANTI	T7 RNA polymerase	1 × 10^−5^ [35]	2.10 ± 0.48	95.4 ± 1.8	88.2 ± 9.0
PTA	phi29	1 × 10^−6^ [34]	10.44 ± 4.83	99.8 ± 0.3	97.3 ± 3.9

The scWGA data were sourced from Chen et al. [15], Pena et al. [16], and this study.

## Data Availability

Raw data have been deposited in the China National Center for Bioinformation with accession number PRJCA055848. Codes for the DIAG and for in silico data generation are available at https://doi.org/10.5281/zenodo.18269102 and https://doi.org/10.5281/zenodo.18383937, respectively.

## Supplementary Materials

The following supporting information can be downloaded at https://www.mdpi.com/article/10.3390/biology15100800/s1, Figure S1: The error probabilities of each type under varying error rates obtained from Monte Carlo simulations; Figure S2: The performance of the DIAG framework in simulation dataset; Figure S3: Performance of the DIAG framework across varying experimental and biological parameters; Figure S4: Benchmarking of SNV calling performance for heterozygous and homozygous sites; Figure S5: Benchmarking of SNV calling performance; Figure S6: The DIA values and distribution of VAF across MALBAC and PTA; Figure S7: The count of variants in amplified samples using MALBAC and PTA; Figure S8: Comparison of DIA values between MALBAC and PTA across multiple datasets; Figure S9: The DIA values and distribution of VAF across multiple methods; Figure S10: The DIA stability across genomic regions with varying GC content; Figure S11: The relationship between DIA estimates and Gini index; Figure S12: The relationship between DIA estimates and sequencing depth; Table S1: The difference in common scWGA methods; Table S2: The sample information used in this study; Table S3: The data information used in this study. References [12,13,14,15,16,17,18,19,38] are cited in the supplementary materials.

## Abbreviations

The following abbreviations are used in this manuscript:

WGA	Whole-Genome Amplification
DIAG	Depth of Independent Amplicons Gauge
DIA	Depth of Independent Amplicons
KL	Kullback–Leibler
scWGA	Single-Cell Whole-Genome Amplification
DOP-PCR	Degenerate Oligonucleotide-Primed PCR
MDA	Multiple Displacement Amplification
MALBAC	Multiple Annealing and Looping-Based Amplification Cycles
LIANTI	Linear Amplification via Transposon Insertion
PTA	Primary Template-directed Amplification
SISSOR	Single-Stranded Sequencing using Microfluidic Reactors
CNV	Copy Number Variation
SNV	Single-Nucleotide Variant
VAF	Variant Allele Frequency
NGS	Next-Generation Sequencing
CV	Coefficient of Variation
PDF	Probability Density Function
CDF	Cumulative Distribution Function
TP	True Positive
FN	False Negative
FP	False Positive
ADO	Allele Dropout
BQSR	Base Quality Score Recalibration
VQSR	Variant Quality Score Recalibration
HTML	Hyper Text Markup Language
SNR	Signal-to-Noise Ratio
MoM	Method of Moments
AFS	Allele Frequency Spectrum
RD	Reference Depth
AD	Alternative Depth
IQR	Inter-Quartile Range
TN	True Negative
SM	Somatic Mutation
GDP	Generic Diagramming Platform
