# Supplementary material for: DIAG: A Framework for Evaluating Whole-Genome Amplification Quality in Single-Cell SNV Analysis
Source: Biology (Basel). 2026 May 18;15(10):800. doi: 10.3390/biology15100800 (PMC13203589; doi:10.3390/biology15100800)
Supplement: Supplementary file 1 [file biology-15-00800-s001.zip › biology-4220454-supplementary.pdf]

Supplementary materials for

DIAG: A Framework for Evaluating Whole-Genome Amplification Quality in Single-Cell SNV Analysis

Di Zhang [1,2,3,†], Mengdong Zhang [3,†], Ao Zhang [3], Siqi Yang [4], Wenfeng Huang [4], Tianqi Cao [3], Xuan Bu [3], Zhan Liu [3], Bingjie Chen [4,*] and Shanjun Deng [3,*]

1 School of Life Science, Jiaying University, Meizhou 514015, China; zhangd256@mail.sysu.edu.cn
2 Conservation and Utilization Laboratory of Mountain Characteristic Resources in Guangdong Province, Meizhou 514015, China
3 MOE Key Laboratory of Gene Function and Regulation, State Key Laboratory of Biocontrol, Innovation Center for Evolutionary Synthetic Biology, School of Life Sciences, Sun Yat-Sen University, Guangzhou 510275, China; zhangmd29@mail2.sysu.edu.cn (M.Z.); awjang@foxmail.com (A.Z.); caotq3@mail.sysu.edu.cn (T.C.); buxuan3@mail2.sysu.edu.cn (X.B.); liuzh526@mail.sysu.edu.cn (Z.L.)
4 The Guangdong-Hong Kong-Macao Joint Laboratory for Cell Fate Regulation and Diseases, GMU-GIBH Joint School of Life Sciences, Guangzhou Medical University, Guangzhou 511436, China; sqyangh@163.com (S.Y.); hwf13414590739@outlook.com (W.H.)
* Correspondence: bingjiechen@gzhmu.edu.cn (B.C.); dengshj8@mail.sysu.edu.cn (S.D.)
† These authors contributed equally to this work.

Contents

Figure S1. The error probabilities of each type under varying error rates obtained from Monte Carlo simulations.
Figure S2. The performance of the DIAG framework in simulation dataset.
Figure S3. Performance of the DIAG framework across varying experimental and biological parameters.
Figure S4. Benchmarking of SNV calling performance for heterozygous and homozygous sites.
Figure S5. Benchmarking of SNV calling performance.
Figure S6. The DIA values and distribution of VAF across MALBAC and PTA.
Figure S7. The count of variants in amplified samples using MALBAC and PTA.
Figure S8. Comparison of DIA values between MALBAC and PTA across multiple datasets.
Figure S9. The DIA values and distribution of VAF across multiple methods.
Figure S10. The DIA stability across genomic regions with varying GC content.
Figure S11. The relationship between DIA estimates and Gini index.
Figure S12. The relationship between DIA estimates and sequencing depth.
Table S1. The difference in common scWGA methods.
Table S2. The sample information used in this study.
Table S3. The data information used in this study.

Supplementary Figures

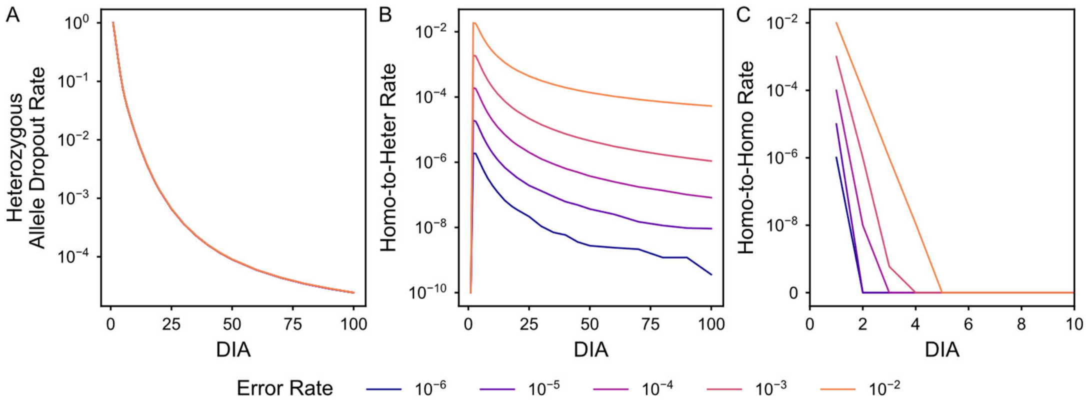

Figure S1. The error probabilities of each type under varying error rates obtained from Monte Carlo simulations. (A). Heterozygous allele dropout rate. The dropout rate exhibits exponential decay as DIA increases and remains independent of the enzymic error rate. (B). Homo-to-Heter rate. Error probabilities decline gradually for DIA $\geqslant 2$. Higher error rates significantly elevate the plateau. (C) Homo-to-Homo rate. Misclassification probabilities drop rapidly to zero as DIA increase, even under conditions of high enzymic error rate conditions.
The x-axis denotes the actual DIA, y-axis denotes the corresponding error probability, and the color represents the error rate from 1×10^{-2} to 1×10^{-6}.

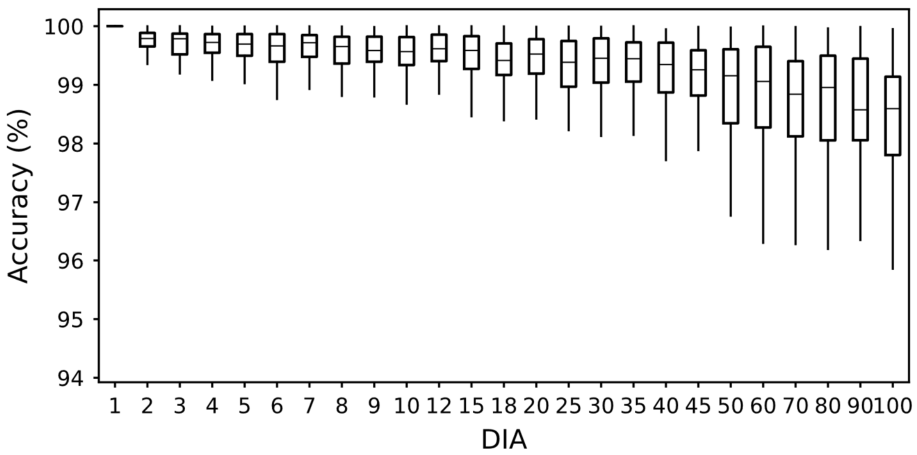

Figure S2. The performance of the DIAG framework in simulation dataset. The DIAG performance remains relatively stable with increasing DIA. The boxplots express the accuracy of DIA estimation with 100 independent replicates. The center line shows the median, the box bounds denote the upper and lower quartile, and the whiskers extend to 1.5x IQR.

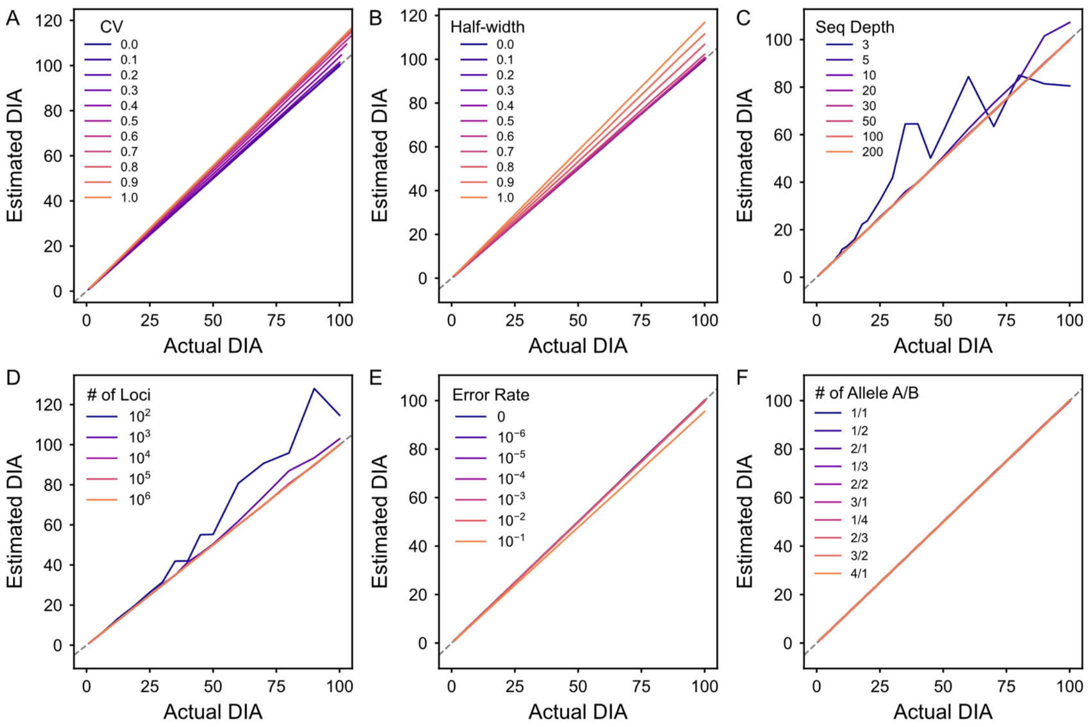

Figure S3. Performance of the DIAG framework across varying experimental and biological parameters. (A). Performance of the DIAG across varying standard deviation of the normal distribution. The DIAG estimation is insensitive to CV under the normal distribution, with color denoting CV values ranging from 0 to 1. (B). Influence of interval width in uniform distribution. The framework performance is unaffected by changes in the relative half-width (δ) from 0 to 1, and color represents different δ values. (C). Sensitivity to sequencing depth. The DIAG performance shows low sensitivity to sequencing depth. The evaluations were performed across sequencing depths of 3x to 200x, with color indicating sequencing depth. (D). Impact of the number of effective loci. More than 1000 effective loci are required for stable performance. Lines indicate performance trends as the number of effective loci scales from 1×10^2 to 1×10^6 and color denotes the number. (E). Robustness to amplification errors. The DIAG performance is decoupled from the amplification error rate. Error rates ranged from 0 to 1e-1, with color representing the error rates. (F). Robustness to CNVs. The estimation is robust across varied ploidy levels, ranging from 2 (diploid) to 5 (pentaploid). The legend notations (Alt/Locus) indicate the copy number of alternative chromosome (Alt, left) and the locus (right), and the colors represent the ploidy levels. In all panels, x-axis represents the ground truth DIA and y-axis denotes the mean DIA estimates derived from 100 independent simulations. The grey dashed diagonal line indicates the $y = x$ identity line, representing a perfect estimation.

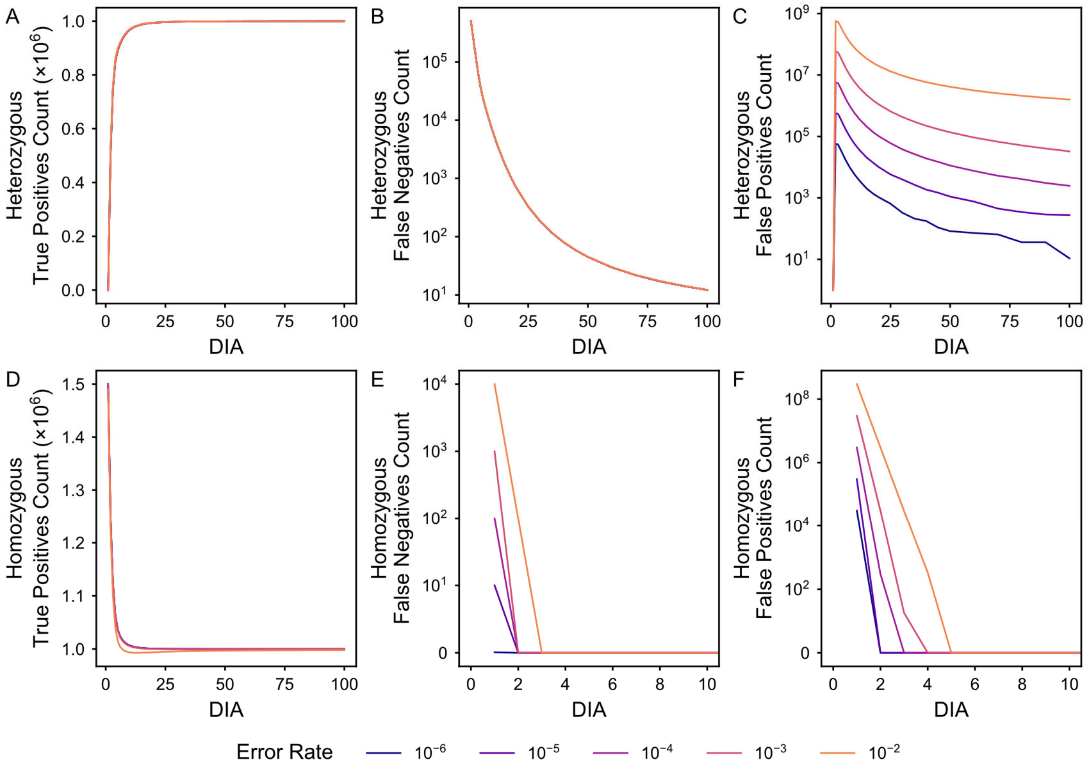

Figure S4. Benchmarking of SNV calling performance for heterozygous and homozygous sites. (A). TP count. The recovery saturates rapidly as DIA increases and remains independent of the enzymic error rate. (B). Heterozygous False FN count. The counts show an exponential decay as DIA increases, consistent across all simulated error conditions. (C). Heterozygous FP count. The values decrease as DIA increases, and are highly sensitive to enzymic error rates, spanning several orders of magnitude. (D). Homozygous TP count. The counts stabilize at the ground-truth value as DIA increases, following an initial peak at low DIA values. (E). Homozygous False FN count. The values drop sharply to zero as DIA increases, with higher error rates requiring slightly higher DIA to reach elimination. (F). Homozygous FP count. The counts decline to zero rapidly as DIA increases, with the baseline error rate significantly influencing the initial magnitude.

Evaluation of TP count, FN count, FP count, under varying DIA values scale from 1-100. The line color indicates enzymic error rates ranging from 1×10^{-6} to 1×10^{-2}.

All metrics were evaluated using *in silico* simulated data under the baseline scenario mimicking human genome (3×10^9 total loci with 1 million heterozygous and 1 million homozygous germline mutation loci).

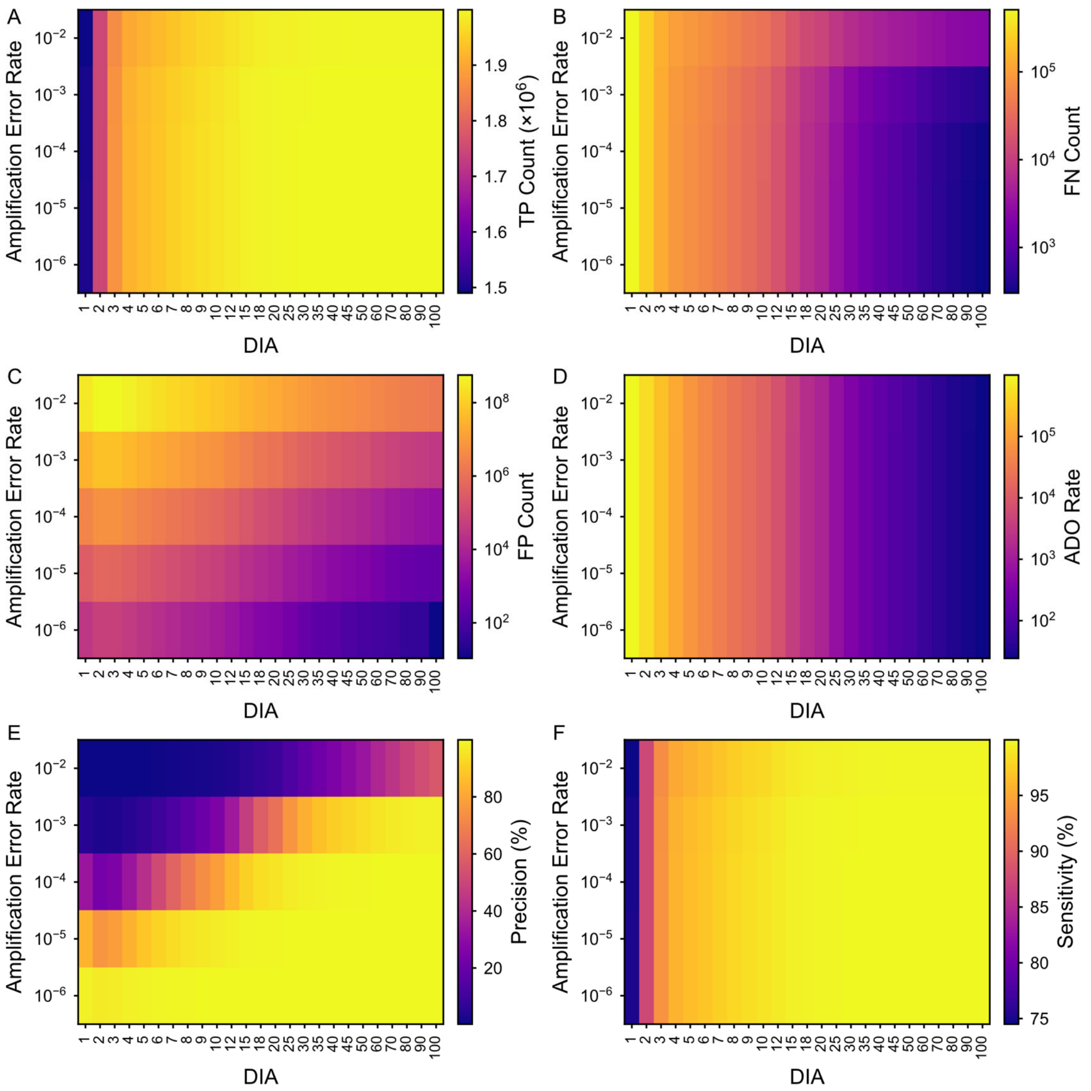

Figure S5. Benchmarking of SNV calling performance. (A-F). Heatmaps displaying core variant calling metrics across a range of DIA values and enzymatic amplification error rates. Metrics shown include TP count (A), ADO rate (B), FN count (C), FP count (D), Precision (E), and Sensitivity (F).

The x-axis represents the DIA ranging from 1 to 100, left y-axis represents the error rate ranging from 1×10^{-6} to 1×10^{-2}, and right y-axis represents the level of metric values.

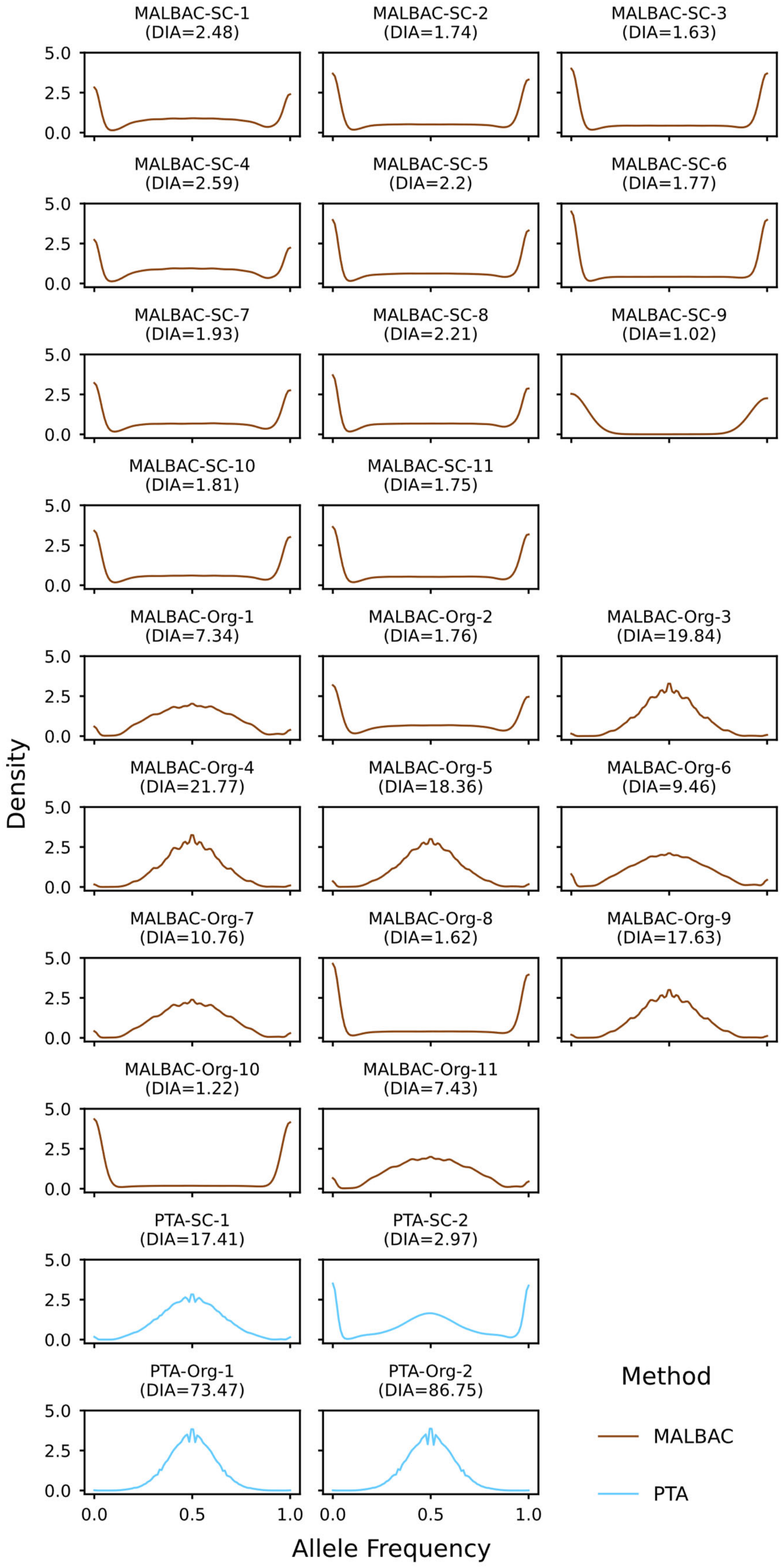
MALBAC-SC-1 (DIA=2.48)
MALBAC-SC-2 (DIA=1.74)
MALBAC-SC-3 (DIA=1.63)
MALBAC-SC-4 (DIA=2.59)
MALBAC-SC-5 (DIA=2.2)
MALBAC-SC-6 (DIA=1.77)
MALBAC-SC-7 (DIA=1.93)
MALBAC-SC-8 (DIA=2.21)
MALBAC-SC-9 (DIA=1.02)
MALBAC-SC-10 (DIA=1.81)
MALBAC-SC-11 (DIA=1.75)
MALBAC-Org-1 (DIA=7.34)
MALBAC-Org-2 (DIA=1.76)
MALBAC-Org-3 (DIA=19.84)
MALBAC-Org-4 (DIA=21.77)
MALBAC-Org-5 (DIA=18.36)
MALBAC-Org-6 (DIA=9.46)
MALBAC-Org-7 (DIA=10.76)
MALBAC-Org-8 (DIA=1.62)
MALBAC-Org-9 (DIA=17.63)
MALBAC-Org-10 (DIA=1.22)
MALBAC-Org-11 (DIA=7.43)
PTA-SC-1 (DIA=17.41)
PTA-SC-2 (DIA=2.97)
PTA-Org-1 (DIA=73.47)
PTA-Org-2 (DIA=86.75)
Density
5.0
2.5
0.0
0.0
0.5
1.0
Allele Frequency
Method
MALBAC
PTA

Figure S6. The DIA values and distribution of VAF across MALBAC and PTA. The DIA values and density distribution of VAFs at heterozygous sites, validated by corresponding bulk tissue sequencing, was characterized for all samples using MALBAC (top) and PTA (bottom). The color represents the method.

A

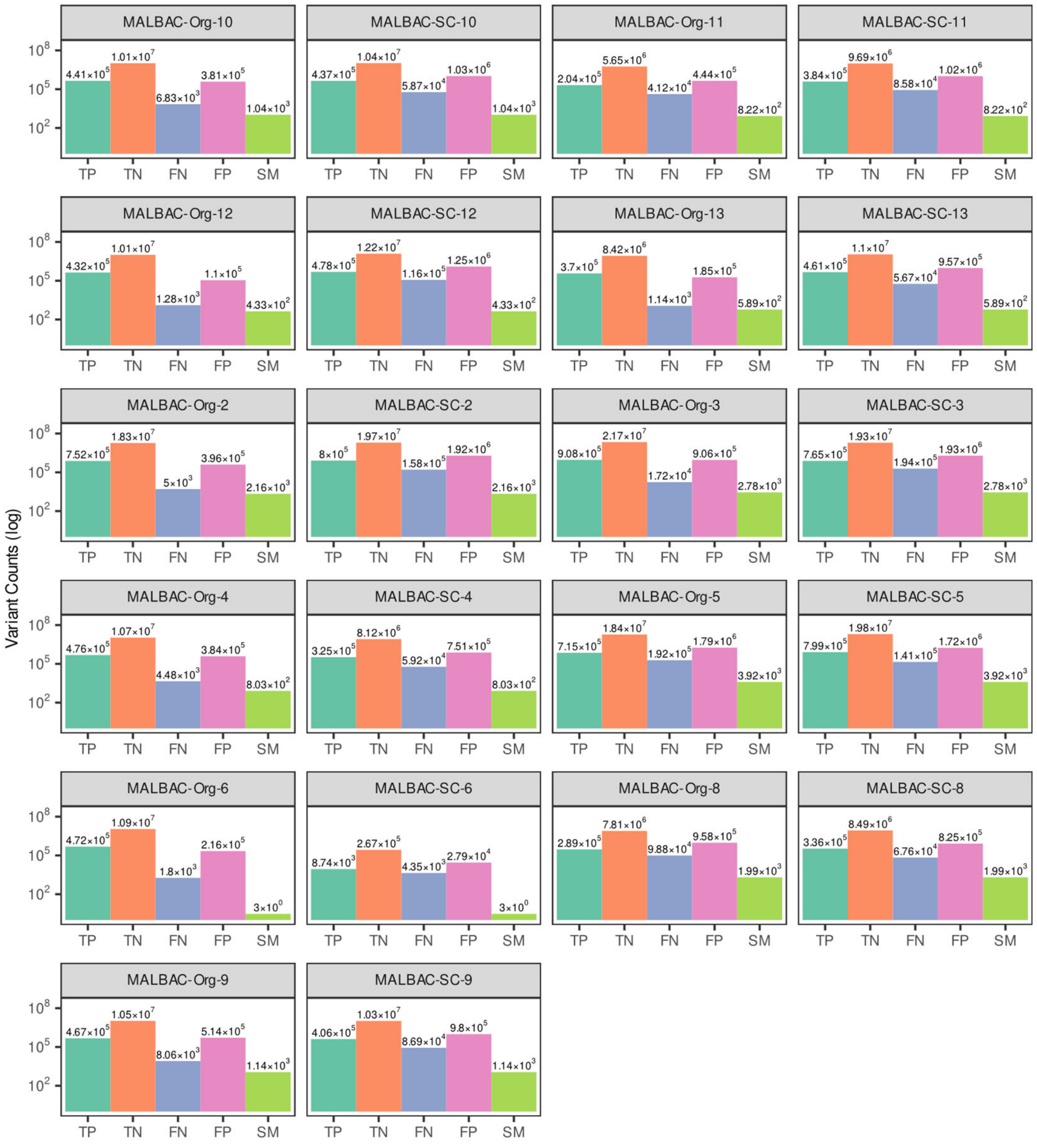

B

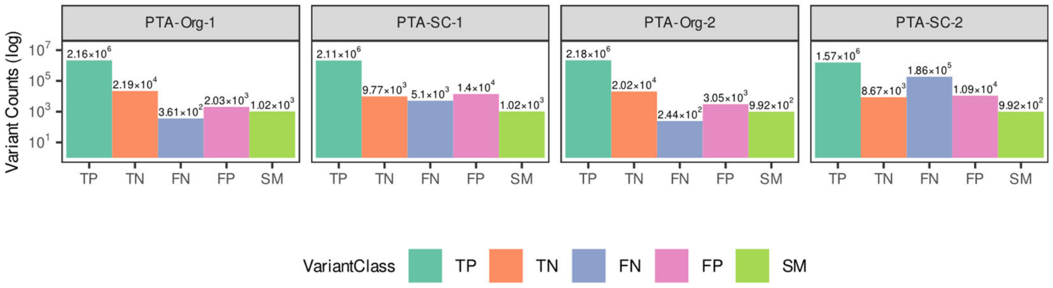

Figure S7. The counts of variants in amplified samples using MALBAC and PTA. (A-B). Mutation counts. The bar plots show the counts of TP, TN, FN, FP of germline variants,

counts of SM amplified by MALBAC (A) and PTA (B). The color represents the different metric.

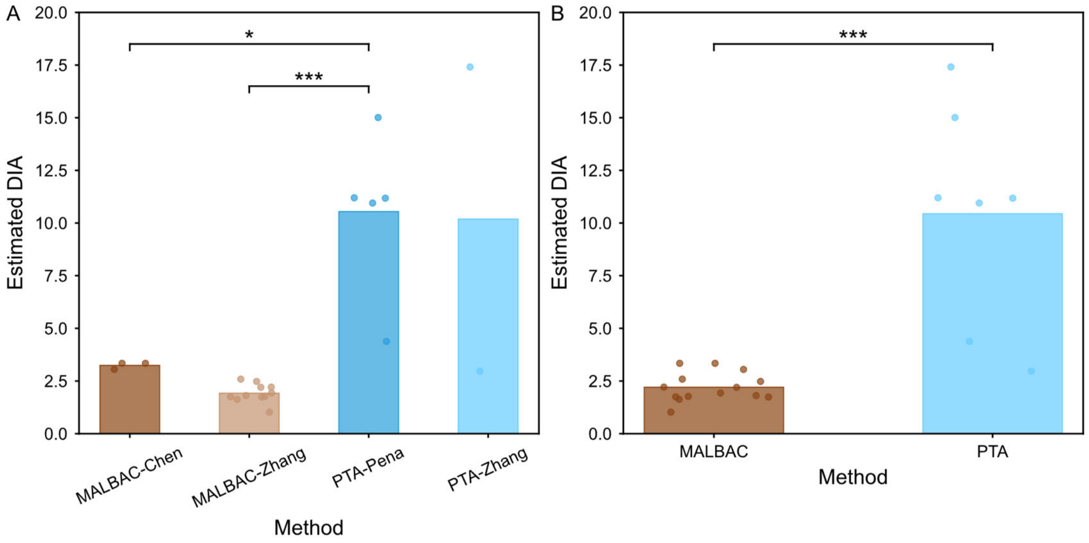

Figure S8. Comparison of DIA values between MALBAC and PTA across multiple datasets. (A). Quantitative comparison of DIA values for MALBAC- amplified single cells (from Chen et al and this study, and PTA-amplified single-cell samples (from Pena et al. and this study). Bars represent the mean DIA values for each group. Statistical significance was determined using the Wilcoxon rank-sum test (*$p < 0.05$, ***$p < 0.001$). (B). Consolidated analysis comparing the overall DIA performance between MALBAC and PTA technologies. Bars represent the mean DIA values for each group. Statistical significance was determined using the Wilcoxon rank-sum test (***$p < 0.001$).

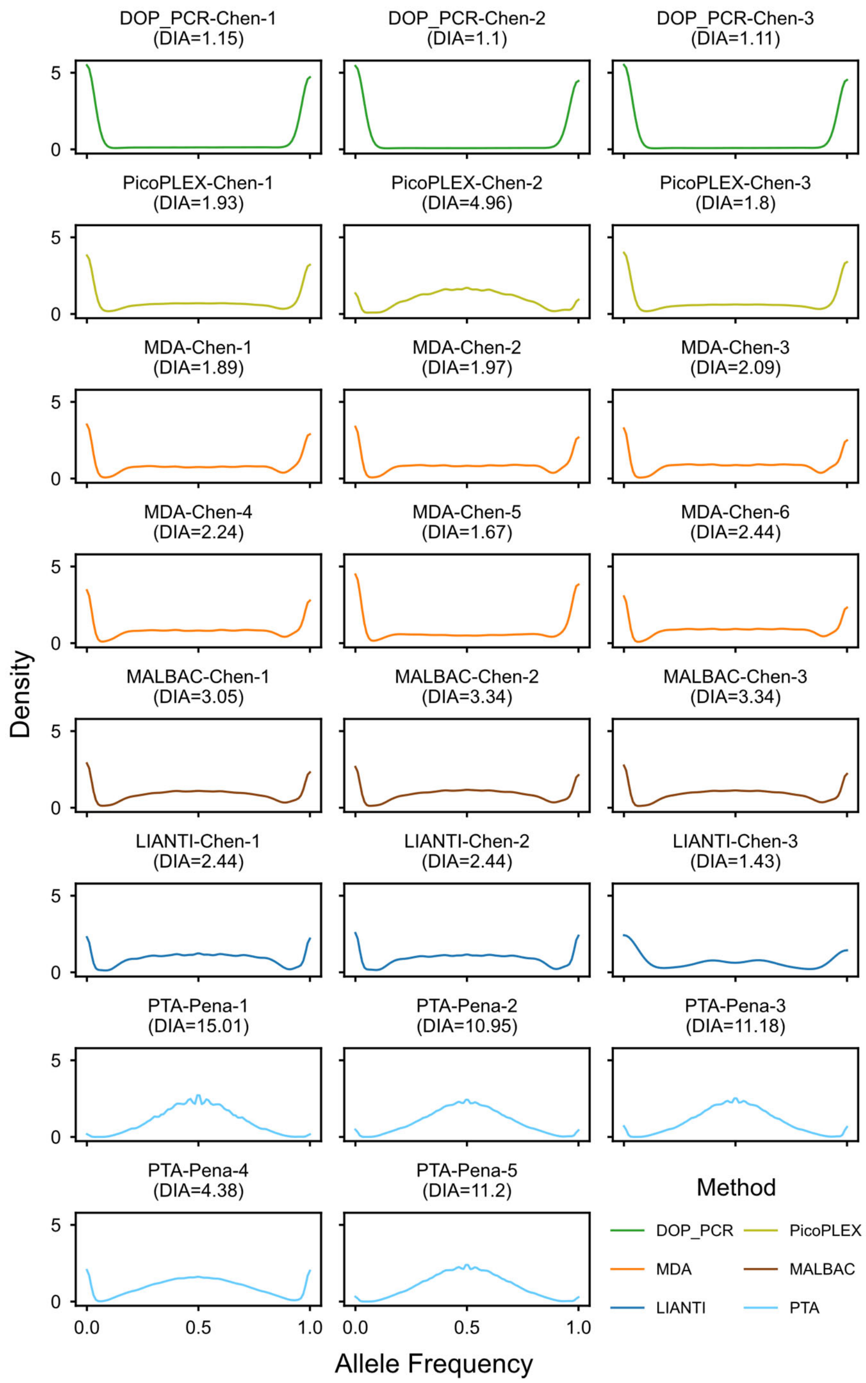

Figure S9. The DIA values and distribution of VAF across multiple methods. The density distribution of VAFs at heterozygous sites for single cell datasets (Chen et al, Pena et al), validated by corresponding bulk tissue sequencing. The color represents the methods. The datasets sourced from Chen et al, Pena et al.

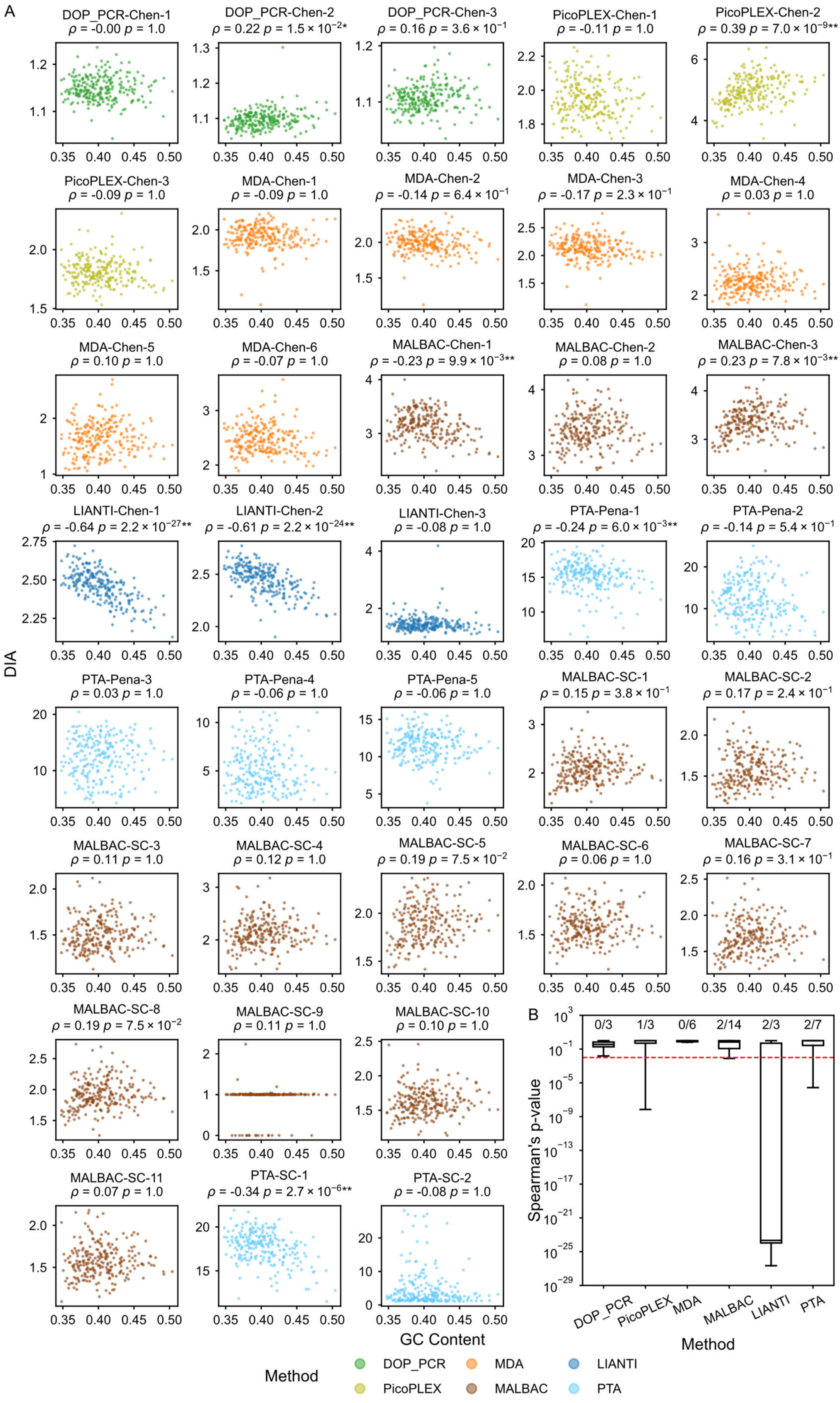

A
DOP_PCR-Chen-1 ρ = -0.00 p = 1.0
DOP_PCR-Chen-2 ρ = 0.22 p = 1.5 × 10^-2*
DOP_PCR-Chen-3 ρ = 0.16 p = 3.6 × 10^-1
PicoPLEX-Chen-1 ρ = -0.11 p = 1.0
PicoPLEX-Chen-2 ρ = 0.39 p = 7.0 × 10^-9**
PicoPLEX-Chen-3 ρ = -0.09 p = 1.0
MDA-Chen-1 ρ = -0.09 p = 1.0
MDA-Chen-2 ρ = -0.14 p = 6.4 × 10^-1
MDA-Chen-3 ρ = -0.17 p = 2.3 × 10^-1
MDA-Chen-4 ρ = 0.03 p = 1.0
MDA-Chen-5 ρ = 0.10 p = 1.0
MDA-Chen-6 ρ = -0.07 p = 1.0
MALBAC-Chen-1 ρ = -0.23 p = 9.9 × 10^-3**
MALBAC-Chen-2 ρ = 0.08 p = 1.0
MALBAC-Chen-3 ρ = 0.23 p = 7.8 × 10^-3**
LIANTI-Chen-1 ρ = -0.64 p = 2.2 × 10^-27**
LIANTI-Chen-2 ρ = -0.61 p = 2.2 × 10^-24**
LIANTI-Chen-3 ρ = -0.08 p = 1.0
PTA-Pena-1 ρ = -0.24 p = 6.0 × 10^-3**
PTA-Pena-2 ρ = -0.14 p = 5.4 × 10^-1
PTA-Pena-3 ρ = 0.03 p = 1.0
PTA-Pena-4 ρ = -0.06 p = 1.0
PTA-Pena-5 ρ = -0.06 p = 1.0
MALBAC-SC-1 ρ = 0.15 p = 3.8 × 10^-1
MALBAC-SC-2 ρ = 0.17 p = 2.4 × 10^-1
MALBAC-SC-3 ρ = 0.11 p = 1.0
MALBAC-SC-4 ρ = 0.12 p = 1.0
MALBAC-SC-5 ρ = 0.19 p = 7.5 × 10^-2
MALBAC-SC-6 ρ = 0.06 p = 1.0
MALBAC-SC-7 ρ = 0.16 p = 3.1 × 10^-1
MALBAC-SC-8 ρ = 0.19 p = 7.5 × 10^-2
MALBAC-SC-9 ρ = 0.11 p = 1.0
MALBAC-SC-10 ρ = 0.10 p = 1.0
MALBAC-SC-11 ρ = 0.07 p = 1.0
PTA-SC-1 ρ = -0.34 p = 2.7 × 10^-6**
PTA-SC-2 ρ = -0.08 p = 1.0
DIA
GC Content
B
0/3
1/3
0/6
2/14
2/3
2/7
Spearman's p-value
DOP_PCR
PicoPLEX
MDA
MALBAC
LIANTI
PTA
Method
Method
DOP_PCR
MDA
LIANTI
PicoPLEX
MALBAC
PTA

Figure S10. The DIA stability across genomic regions with varying GC content. (A). Correlation between estimated DIA and GC content across diverse single-cell libraries. Each scatter plot represents an individual cell, with DIA values calculated for 10 Mb genomic bins (y-axis) plotted against their corresponding GC content (x-axis). Datasets encompass multiple WGA chemistries, including DOP-PCR, PicoPLEX, MDA, MALBAC, LIANTI, and PTA. Spearman's correlation coefficient (ρ) and the associated adjusted p-value are indicated for each cell (* $p < 0.05$, ** $p<0.01$). The majority of samples across different methods show stable DIA estimates regardless of local GC variations. (B). Distribution of Spearman's p-values across different WGA methods. The boxplot summarizes the range of adjusted p-values for the correlation between DIA and GC content within each method. The dashed red line indicates the significance threshold ($p = 0.05$). Fractions above the bars represent the number of cells exhibiting no statistically significant correlations ($p > 0.05$) over the total number of cells tested for each method.

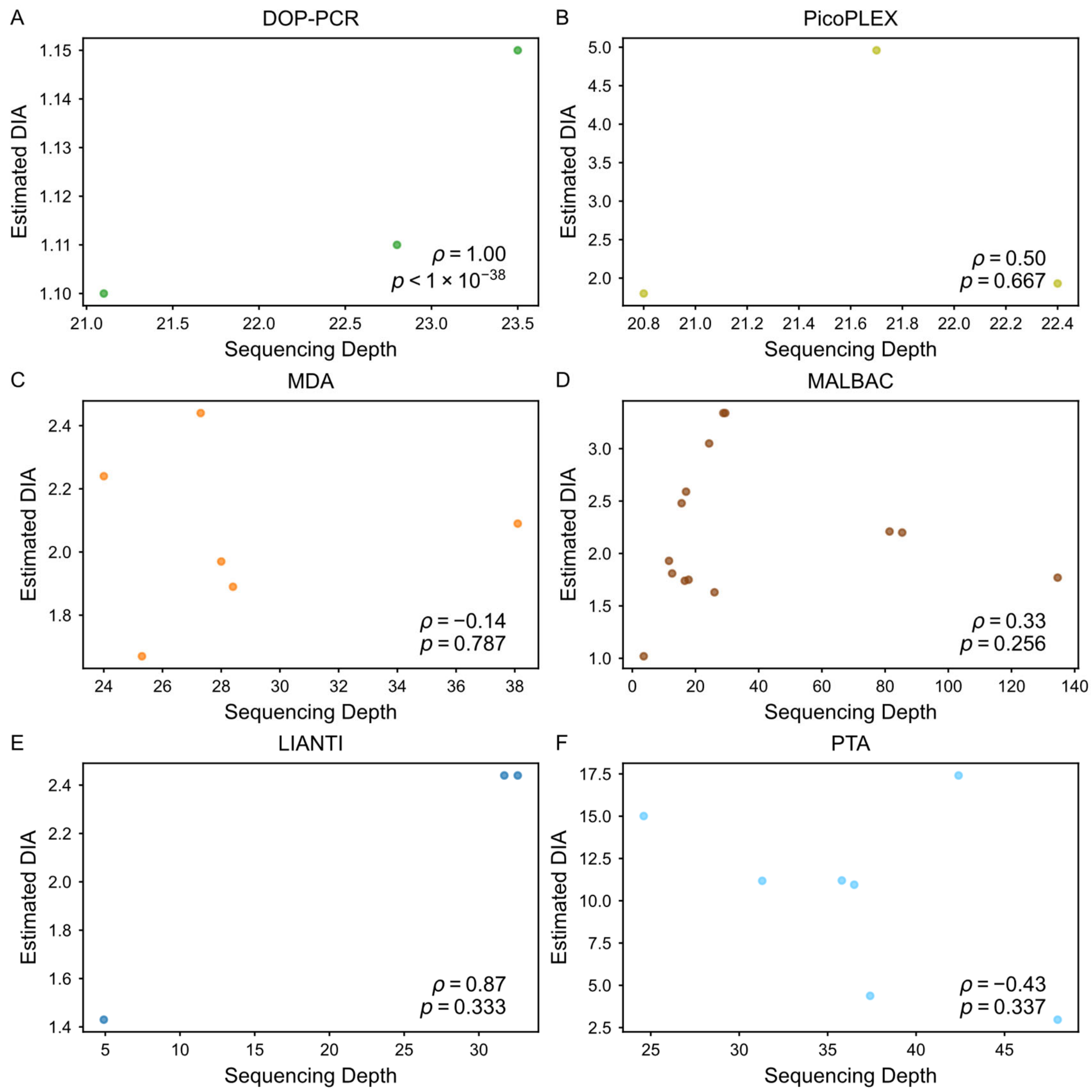

Figure S11. The relationship between DIA estimates and sequencing depth. **(**A-F). Individual data points represent single-cell libraries, with sequencing depth (sequencing depth, x-axis) plotted against the corresponding DIA estimate (y-axis) for DOP-PCR (A), PicoPLEX (B), MDA (C), MALBAC (D), LIANTI (E) and PTA (F). Spearman's correlation coefficient (ρ) and the associated p-value are indicated for each method. In all major WGA methods with a sufficient sample size (e.g., MDA, MALBAC, and PTA), no statistically significant correlation was observed ($p > 0.05$), showing that DIA is an intrinsic indicator of library complexity that is decoupled from sequencing effort.

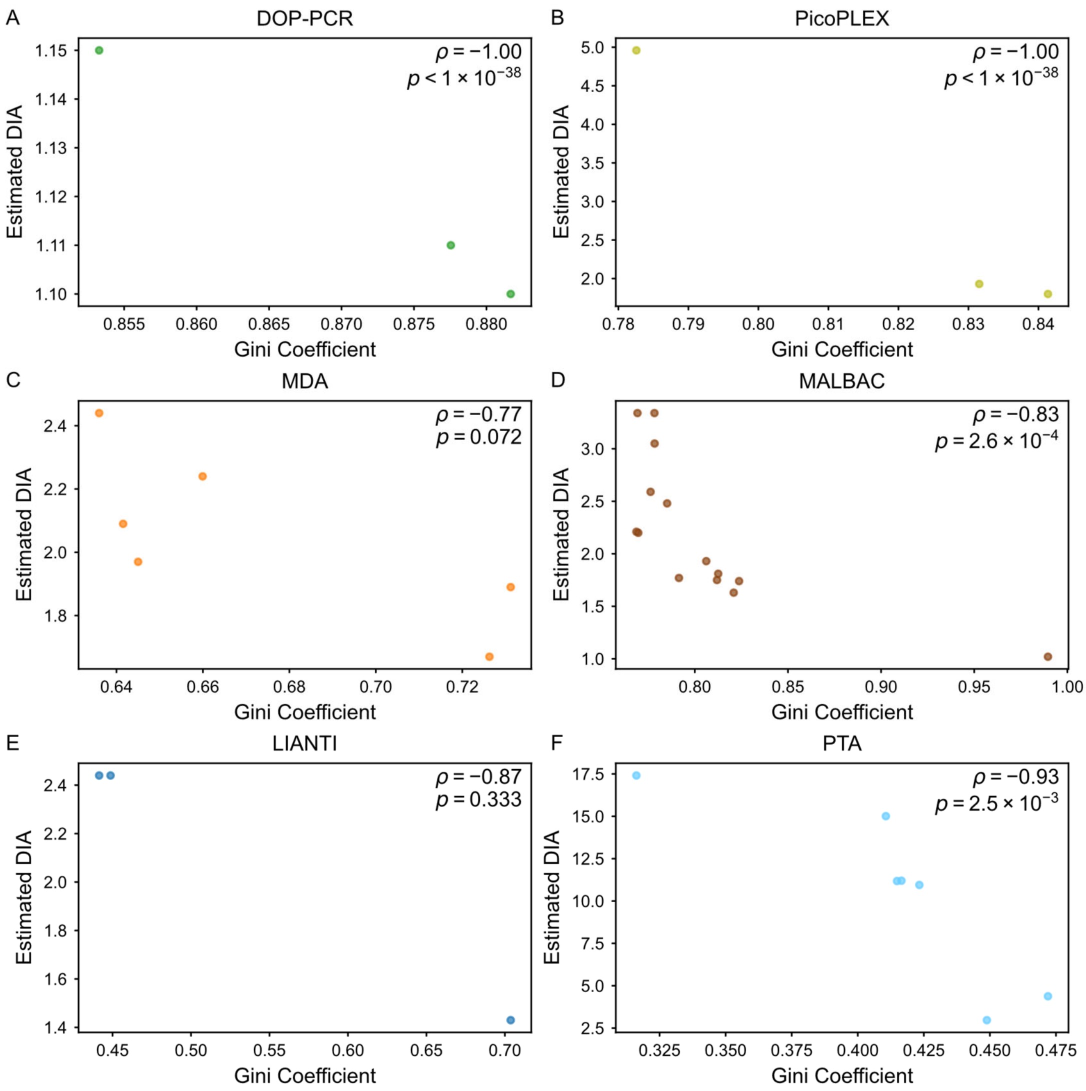

Figure S12. The relationship between DIA estimates and Gini index. (A-F). Individual data points represent single-cell libraries, with coverage-based metric (Gini coefficient, x-axis) plotted against the corresponding DIA estimate (y-axis) for DOP-PCR (A), PicoPLEX (B), MDA (C), MALBAC (D), LIANTI (E) and PTA (F). Spearman's correlation coefficient (ρ) and the associated p-value are indicated for each method.

Supplementary Tables

Table S1. The difference in common scWGA methods.

Method	Amplification process	Amplification Enzyme	Reference	Commercial Kit
DOP-PCR	Partially random primer for a two-step PCR amplification of template genomic DNA	DNA polymerase	[1]	Sigma GenomePlex Single Cell WGA Kit, PerkinElmer DOPlify WGA kit
MDA	Isothermal amplification at 30°C, random hexamer priming and strong strand displacement	phi29	[2]	Qiagen REPLI-g Single Cell Kit，GE Healthcare GenomiPhi DNA Amplification Kit, AmpliQ Genomic Amplifier Kit
MALBAC	Quasilinear amplification by looping structure prevents exponential bias, followed by PCR	Bst, DNA polymerase	[3]	Yikon Genomics MALBAC Single Cell WGA Kit
LIANTI	Tn5 inserts T7 promoter, in vitro transcription to RNA, reverse transcription, linear amplification	T7 RNA polymerase	[4]	NA
TruePrime	TthPrimPol generates primers in situ, phi29 extends, exponential MDA without random primers	TthPrimPol, phi29	[5]	Sygnis TruePrime WGA kit
PicoPLEX	Strand-displacement enzyme, forming hysical hairpin structure, followed high-fidelity PCR enzyme	Strand-displacement enzyme, DNA polymerase	[6]	Rubicon Genomics PicoPLEX WGA Kit, TakaRa PicoPLEX Single Cell WGA Kit
SISSOR	Long single strands physically separated; each strand MDA-amplified independently	phi29, DNA polymerase	[7]	NA
PTA	Modified MDA chemistry reduces runaway amplification, broad size distribution, reduced bias	phi29, DNA polymerase	[8]	BioSkryb ResolveDNA/OME WGA kit
META-CS	Fragmentation, end-tagging both strands, PCR amplification of tagged fragments	Q5 DNA polymerase	[9]	NA

Table S2. The sample information used in this study.

Source	Method	SampleName	Type	CellType	Origin	Donor
Zhang	No-amp	P1-Bulk	Bulk	NA	Lung	P1
Zhang	MALBAC	P1-Org-10	Org	NA	Lung	P1
Zhang	MALBAC	P1-Org-11	Org	NA	Lung	P1
Zhang	MALBAC	P1-Org-12	Org	NA	Lung	P1
Zhang	MALBAC	P1-Org-13	Org	NA	Lung	P1
Zhang	MALBAC	P1-Org-2	Org	NA	Lung	P1
Zhang	MALBAC	P1-Org-3	Org	NA	Lung	P1
Zhang	MALBAC	P1-Org-4	Org	NA	Lung	P1
Zhang	MALBAC	P1-Org-5	Org	NA	Lung	P1
Zhang	MALBAC	P1-Org-6	Org	NA	Lung	P1
Zhang	MALBAC	P1-Org-8	Org	NA	Lung	P1
Zhang	MALBAC	P1-Org-9	Org	NA	Lung	P1
Zhang	MALBAC	P1-SC-10	SC	NA	Lung	P1
Zhang	MALBAC	P1-SC-11	SC	NA	Lung	P1
Zhang	MALBAC	P1-SC-12	SC	NA	Lung	P1
Zhang	MALBAC	P1-SC-13	SC	NA	Lung	P1
Zhang	MALBAC	P1-SC-2	SC	NA	Lung	P1
Zhang	MALBAC	P1-SC-3	SC	NA	Lung	P1
Zhang	MALBAC	P1-SC-4	SC	NA	Lung	P1
Zhang	MALBAC	P1-SC-5	SC	NA	Lung	P1
Zhang	MALBAC	P1-SC-6	SC	NA	Lung	P1
Zhang	MALBAC	P1-SC-8	SC	NA	Lung	P1
Zhang	MALBAC	P1-SC-9	SC	NA	Lung	P1
Zhang	No-amp	P2-Bulk	Bulk	NA	Lung	P2
Zhang	PTA	P2-Org-1	Org	NA	Lung	P2
Zhang	PTA	P2-Org-2	Org	NA	Lung	P2
Zhang	PTA	P2-SC-1	SC	NA	Lung	P2
Zhang	PTA	P2-SC-2	SC	NA	Lung	P2
Pena	No-amp	cd34_bulk	SC	NA	Cord Bood	NA
Pena	PTA	cd34_cell1	SC	cd34	Cord Bood	NA
Pena	PTA	cd34_cell2	SC	cd34	Cord Bood	NA
Pena	PTA	cd34_cell3	SC	cd34	Cord Bood	NA
Pena	PTA	cd34_cell4	SC	cd34	Cord Bood	NA
Pena	PTA	cd34_cell5	SC	cd34	Cord Bood	NA
Chen	No-amp	Bulk1	Bulk	NA	Cell Line	NA
Chen	LIANTI	LIANTI_BJ1	SC	BJ	Cell Line	NA
Chen	LIANTI	LIANTI_BJ2	SC	BJ	Cell Line	NA
Chen	LIANTI	LIANTI_BJ3	SC	BJ	Cell Line	NA
Chen	MDA	MDA_Q1	SC	BJ	Cell Line	NA
Chen	MDA	MDA_Q5	SC	BJ	Cell Line	NA
Chen	MDA	MDA_Q9	SC	BJ	Cell Line	NA
Chen	MALBAC	MALBAC_YK1	SC	BJ	Cell Line	NA
Chen	MALBAC	MALBAC_YK2	SC	BJ	Cell Line	NA
Chen	MALBAC	MALBAC_YK5	SC	BJ	Cell Line	NA
Chen	DOP-PCR	DOP_PCR_S3	SC	BJ	Cell Line	NA
Chen	DOP-PCR	DOP_PCR_S4	SC	BJ	Cell Line	NA
Chen	DOP-PCR	DOP_PCR_S5	SC	BJ	Cell Line	NA
Chen	MDA	MDA_GE10	SC	BJ	Cell Line	NA
Chen	MDA	MDA_GE2	SC	BJ	Cell Line	NA
Chen	MDA	MDA_GE4	SC	BJ	Cell Line	NA
Chen	PicoPLEX	PicoPLEX_R3	SC	BJ	Cell Line	NA
Chen	PicoPLEX	PicoPLEX_R7	SC	BJ	Cell Line	NA
Chen	PicoPLEX	PicoPLEX_R9	SC	BJ	Cell Line	NA

Table S3. The data information used in this study.

SampleName	Platform	MappingRate(%)	TotalSeqs(M)	DuplicationRate(%)	GCcontent(%)	Sequencing Depth(X)	MedianCoverage(X)
P1-Bulk	illumina-NovaSeq6000/X plus	99.9	1223	6.9	42.8	59.2	35
P1-Org-10	illumina-NovaSeq6000/X plus	99.6	334	11.1	46.9	16.2	3
P1-Org-11	illumina-NovaSeq6000/X plus	99.2	153.3	2	44.6	7.4	1
P1-Org-12	illumina-NovaSeq6000/X plus	99.6	364.5	5	47.5	17.6	4
P1-Org-13	illumina-NovaSeq6000/X plus	99.7	297.9	10.7	47.8	14.4	3
P1-Org-2	illumina-NovaSeq6000/X plus	98.8	1502.5	7.7	49	72.7	12
P1-Org-3	illumina-NovaSeq6000/X plus	98.8	2242.1	10.8	48.9	108.5	18
P1-Org-4	illumina-NovaSeq6000/X plus	99.7	346.2	9.6	47.2	16.8	4
P1-Org-5	illumina-NovaSeq6000/X plus	98.9	1712.2	5.9	45	82.8	8
P1-Org-6	illumina-NovaSeq6000/X plus	99.5	378.7	10.3	46.5	18.3	4
P1-Org-8	illumina-NovaSeq6000/X plus	99	397.5	5.1	42.8	19.2	0
P1-Org-9	illumina-NovaSeq6000/X plus	99.6	339.5	11.5	46.9	16.4	3
P1-SC-10	illumina-NovaSeq6000/X plus	99.3	322.7	5.4	44.7	15.6	2
P1-SC-11	illumina-NovaSeq6000/X plus	99.2	342.8	4.3	43.8	16.6	1
P1-SC-12	illumina-NovaSeq6000/X plus	99.3	537.7	5.8	44.4	26	2
P1-SC-13	illumina-NovaSeq6000/X plus	99.3	352	4.5	44.7	17	2
P1-SC-2	illumina-NovaSeq6000/X plus	99	1765.5	8	45.5	85.4	11

P1-SC-3	illumina-NovaSeq6000/X plus	98.8	2781.3	12.6	45.4	134.6	11
P1-SC-4	illumina-NovaSeq6000/X plus	99.2	240.4	4	44.7	11.6	1
P1-SC-5	illumina-NovaSeq6000/X plus	98.9	1682.6	6.1	46.2	81.4	10
P1-SC-6	illumina-NovaSeq6000/X plus	93.6	74	9.7	46.6	3.6	0
P1-SC-8	illumina-NovaSeq6000/X plus	99.2	260.6	4.6	44.2	12.6	1
P1-SC-9	illumina-NovaSeq6000/X plus	99.2	368.1	5.1	44.3	17.8	2
P2-Bulk	illumina-NovaSeq6000/X plus	99.8	1752.1	26.6	40.2	84.8	58
P2-Org-1	illumina-NovaSeq6000/X plus	99.7	853.7	22.1	39.3	41.3	27
P2-Org-2	illumina-NovaSeq6000/X plus	99.9	777.9	24.2	39.9	37.6	24
P2-SC-1	illumina-NovaSeq6000/X plus	99.4	876.2	24.6	40.8	42.4	24
P2-SC-2	illumina-NovaSeq6000/X plus	99.4	991.1	26.5	40.5	48	24
cd34_bulk	illumina-NovaSeq6000	100	1310.4	11	42.4	63.4	54
cd34_cell1	illumina-NovaSeq6000	99.9	509.4	13.7	40.8	24.6	17
cd34_cell2	illumina-NovaSeq6000	99.9	755	13.4	41	36.5	25
cd34_cell3	illumina-NovaSeq6000	99.9	646.7	14.4	40.9	31.3	21
cd34_cell4	illumina-NovaSeq6000	99.9	773.5	22.5	40.8	37.4	20
cd34_cell5	illumina-NovaSeq6000	99.9	739.1	15.4	40.9	35.8	24
Bulk1	illumina-Hiseq2500	97.3	911.1	7.6	41.1	44.1	35
LIANTI_BJ1	illumina-Hiseq2500	97.2	674.2	8.7	41.8	32.6	16
LIANTI_BJ2	illumina-Hiseq2500	98.5	654.7	8.3	41.6	31.7	18
LIANTI_BJ3	illumina-Hiseq2500	90.1	101.9	6	42	4.9	1
MDA_Q1	illumina-Hiseq2500	100	495.3	6.7	46.7	24	10
MDA_Q5	illumina-Hiseq2500	100	522.4	7.3	46.1	25.3	7

MDA_Q9	illumina-Hiseq2500	100	563.7	11	46.4	27.3	12
MALBA_YK1	illumina-Hiseq2500	99.1	503	7.3	48.2	24.3	5
MALBAC_YK2	illumina-Hiseq2500	99.2	594.2	11.3	48.2	28.8	5
MALBAC_YK5	illumina-Hiseq2500	99.5	607.6	11.5	47.1	29.4	6
DOP_PCR_S3	illumina-Hiseq2500	99.9	486.6	8	43.5	23.5	2
DOP_PCR_S4	illumina-Hiseq2500	99.9	436.6	8.1	44	21.1	1
DOP_PCR_S5	illumina-Hiseq2500	99.8	470.5	8.1	43.8	22.8	1
MDA_GE10	illumina-Hiseq2500	99.9	587.4	9.2	45.2	28.4	6
MDA_GE2MDA_GE4	illumina-Hiseq2500	99.3	788.3	11.1	44.8	38.1	11
PicoPLEX_R3	illumina-Hiseq2500	99.9	463.3	7	48.5	22.4	3
PicoPLEX_R7	illumina-Hiseq2500	99.9	448.9	7.1	47.7	21.7	5
PicoPLEX_R9	illumina-Hiseq2500	99.8	430.7	6.6	48.7	20.8	3

Reference

1. Telenius, H.; Carter, N.P.; Bebb, C.E.; Nordenskjöld, M.; Ponder, B.A.; Tunnacliffe, A. Degenerate Oligonucleotide-Primed PCR: General Amplification of Target DNA by a Single Degenerate Primer. *Genomics* **1992**, *13*, 718–725, doi:10.1016/0888-7543(92)90147-k.
2. Dean, F.B.; Hosono, S.; Fang, L.; Wu, X.; Faruqi, A.F.; Bray-Ward, P.; Sun, Z.; Zong, Q.; Du, Y.; Du, J.; et al. Comprehensive Human Genome Amplification Using Multiple Displacement Amplification. *Proc Natl Acad Sci U S A* **2002**, *99*, 5261–5266, doi:10.1073/pnas.082089499.
3. Zong, C.; Lu, S.; Chapman, A.R.; Xie, X.S. Genome-Wide Detection of Single-Nucleotide and Copy-Number Variations of a Single Human Cell. *Science* **2012**, *338*, 1622–1626, doi:10.1126/science.1229164.
4. Chen, C.; Xing, D.; Tan, L.; Li, H.; Zhou, G.; Huang, L.; Xie, X.S. Single-Cell Whole-Genome Analyses by Linear Amplification via Transposon Insertion (LIANTI). *Science* **2017**, doi:10.1126/science.aak9787.
5. Picher, Á.J.; Budeus, B.; Wafzig, O.; Krüger, C.; García-Gómez, S.; Martínez-Jiménez, M.I.; Díaz-Talavera, A.; Weber, D.; Blanco, L.; Schneider, A. TruePrime Is a Novel Method for Whole-Genome Amplification from Single Cells Based on TthPrimPol. *Nat Commun* **2016**, *7*, 13296, doi:10.1038/ncomms13296.
6. Langmore, J.P. Rubicon Genomics, Inc. *Pharmacogenomics* **2002**, *3*, 557–560, doi:10.1517/14622416.3.4.557.
7. Chu, W.K.; Edge, P.; Lee, H.S.; Bansal, V.; Bafna, V.; Huang, X.; Zhang, K. Ultraaccurate Genome Sequencing and Haplotyping of Single Human Cells. *Proc Natl Acad Sci U S A* **2017**, *114*, 12512–12517, doi:10.1073/pnas.1707609114.
8. Gonzalez-Pena, V.; Natarajan, S.; Xia, Y.; Klein, D.; Carter, R.; Pang, Y.; Shaner, B.; Annu, K.; Putnam, D.; Chen, W.; et al. Accurate Genomic Variant Detection in Single Cells with Primary Template-Directed Amplification. *Proceedings of the National Academy of Sciences* **2021**, *118*, e2024176118, doi:10.1073/pnas.2024176118.
9. Xing, D.; Tan, L.; Chang, C.-H.; Li, H.; Xie, X.S. Accurate SNV Detection in Single Cells by Transposon-Based Whole-Genome Amplification of Complementary Strands. *Proceedings of the National Academy of Sciences* **2021**, *118*, e2013106118, doi:10.1073/pnas.2013106118.
